# STRategy: A support system for collecting and analyzing next-generation sequencing data of short tandem repeats for forensic science

**DOI:** 10.1371/journal.pone.0282551

**Published:** 2023-07-17

**Authors:** Nuttachai Kulthammanit, Tikumphorn Sathirapatya, Poonyapat Sukawutthiya, Hasnee Noh, Kornkiat Vongpaisarnsin, Duangdao Wichadakul

**Affiliations:** 1 Department of Computer Engineering, Faculty of Engineering, Chulalongkorn University, Bangkok, Thailand; 2 Department of Forensic Medicine, Faculty of Medicine, Chulalongkorn University, Bangkok, Thailand; 3 Forensic Serology and DNA, King Chulalongkorn Memorial Hospital and Thai Red Cross Society, Bangkok, Thailand; 4 Forensic Genetics Research Unit, Ratchadapiseksompotch Fund, Faculty of Medicine, Chulalongkorn University, Bangkok, Thailand; 5 Center of Excellence in Systems Biology, Faculty of Medicine, Chulalongkorn University, Bangkok, Thailand; Universiti Teknologi Malaysia - Main Campus Skudai: Universiti Teknologi Malaysia, MALAYSIA

## Abstract

Short tandem repeats (STRs) are short repeated sequences commonly found in the human genome and valuable in forensic science, used for human identity and relatedness markers. Next-generation sequencing (NGS) technologies, e.g., ForenSeq Signature Prep, can sequence STRs, inferring length-based alleles and single nucleotide polymorphisms (SNPs) and providing valuable insights into population and sub-population structures. Despite the potential benefits of NGS for STRs, no open-source software platform integrates the collection, management, and analysis of STR data from NGS into one place. Users must use multiple programs to process their STR data and then collect the results into a separate database or a file system folder. Moreover, analyzing repeat structures (STR repeat motifs) may require learning multiple software tools, making the process inefficient and cumbersome. To address this gap, we introduce the STRategy, a standalone web-based application supporting essential STR data management and analysis capabilities. The STRategy allows users to collect their data into its database, automatically calculates forensic parameters, and visualizes the analyzed data in various forms. Users can search the database using different options, such as by profile, loci, and genotypes, with and without a specific test kit. Moreover, users can also find the nucleotide variants of a locus among the samples. We designed the STRategy for internal use in a laboratory or an organization. Hence, our system includes role-based access control that allows users to search for or access specific data based on their responsibilities. The administrator role can customize the system, for example, configure maps according to the samples’ geographic data, and manage reference STR repeat motifs. A laboratory or an organization can download and install a copy of STRategy on their local system using Docker, as described in https://github.com/cucpbioinfo/STRategy. In summary, the STRategy is an end-to-end system that provides users with a database to collect the analyzed STR data from NGS, the dynamic analyses of forensic parameters, and the variants of STR patterns according to the newly added samples, which are then explorable via various search options and visualizations. The system is helpful for both forensic investigations and forensic genetics.

## Introduction

Short tandem repeats or short repeated Deoxyribonucleic acid (DNA) sequences are repeating units of DNA sequences commonly used in forensics, genetic mapping, and evolutionary studies [1]. STRs have a notable application in forensic genetics due to their use as an individualized DNA marker which could be applied to criminal cases or relatedness. Forensic STR databases have revolutionized the field of forensic science, enabling investigators to solve crimes more quickly and accurately. The databases are used in various forensic investigations, including sexual assault cases, homicides, and missing persons. Forensic experts can also use these databases to identify individuals in mass disasters, such as airplane crashes or natural disasters. Most recent databases have been developed using STRs based on capillary electrophoresis (STR-CE) [2] that defined an allele from a number of repeat units. With the advent of next-generation sequencing (NGS) [3] technologies, the analysis of STRs has become more efficient and accurate. In addition, NGS can generate high-throughput data that can be used to analyze large numbers of STR loci in a single run, providing more precise and reliable results. In forensic genetics, one of the key advantages of NGS-based STR analysis is the ability to generate large amounts of high-quality data, which can be used to create population databases. While the conventional method of STR-CE detection provides information on the length of alleles, NGS can improve the precision and identify variants in the nucleotide sequence within the repeat regions and the nearby flanking regions [4–6]. Various specialized software tools have been developed to align the NGS-generated STR data and analyze the results, such as STRait Razor 3.0 [7] and lobSTR [8], which offer different approaches to handle the complexities and heterogeneities of the data.

Analyzing sequence-based STR data from NGS for forensic science requires users to learn multiple tools, such as STRAF [9] for population parameter analysis, STRait Razor for characterizing alleles and repeat structure, and OmniPop200.1 [10] for profile searching. Additionally, some software tools require data conversion to a specific format, adding to the complexity and time required. Recognizing these challenges, we propose the STRategy, a web-based application that conveniently collects and analyzes the sequence-based STR data from the ForenSeq sample detail report [11]. The system serves as a database with a management system for STR data, which can be used within laboratories and organizations. Furthermore, it provides essential analyzing tools to analyze STR data in the database and visualizations to display results.

## Materials and methods

### Overview

We designed the STRategy as a web-based application that allows users to organize and analyze STR data efficiently and conveniently. The following subsections describe the underlying data and core methods designed and developed within the system.

### The short tandem repeat data

The STRategy accepts STR data generated from the ForenSeq Universal Analysis Software v1.3 called ForenSeq sample detail report; an Excel file with nine sheets, namely Autosomal STRs, Autosomal STR Figure, Y STRs, Y STR Figure, X STRs, X STR Figure, iSNPs, iSNP Figure, and Settings described as follows.

**Fig 1:**
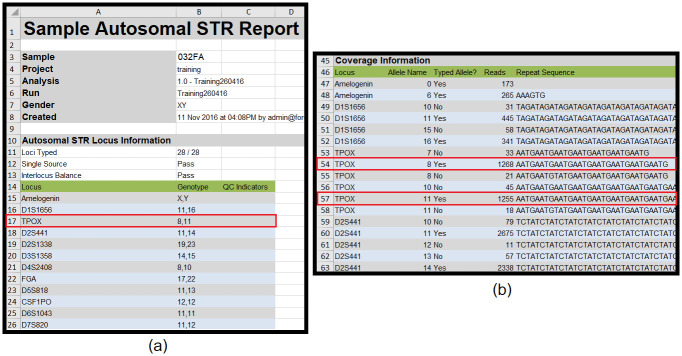
Autosomal STR data sheet from next-generation sequencing (ForenSeq). (a) STR genotype (b) STR allele sequence information.

- Autosomal STRs, Y STRs, and X STRs sheets–contain the length-based allele table (Fig 1a) and the sequence-based allele table (Fig 1b). The length-based allele table contains genotypes for each locus. These genotypes are derived from the sequences of that genotype in the sequence-based allele table. Only the rows in the sequence-based allele table with a "Yes" value in the "Typed Allele" column are used to derive the genotypes in the length-based allele table. For example, the locus TPOX (row 17 of Fig 1a) having genotype 8,11 in the length-based allele table has sequence detail from rows 54 and 57 of Fig 1b in the sequence-based allele table.
- iSNPs sheet–contains tables of single nucleotide polymorphism genotypes and SNP allele read coverage information for each locus (S1 Fig). This sheet resembles the Autosomal STRs, Y STRs, and X STRs sheets. The first table is the SNP genotype, and the second is the SNP allele information. The STRategy will focus on only these two tables.
- Autosomal STR Figure, Y STR Figure, X STR Figure, and iSNP Figure sheets–contain a summary table of read counts for each locus and a bar chart representing the overall read count. The STRategy does not collect data from these four sheets.
- Settings sheet–contains all the constants used in the experiment. The STRategy also does not collect any information from this sheet.

The STR data in the ForenSeq sample detail report usually does not have a flanking region. There is another file called the flanking report, which is not used by the system. However, some markers have flanking regions in the sample details report—for example, D1S1656 and D5S818. This file does not include personal information, e.g., gender and race. All samples’ STR data are saved in the database with sample IDs that will be automatically linked to personal data when uploaded into the system by laboratory users.

The showcase STRategy contains 125 mocked samples constructed by swapping genotypes in the same locus among 125 actual ForenSeq sample detail reports. The original data have been anonymized to protect the privacy of the individuals they came from, and the mocked samples cannot be converted back to the original data. It is important to note that the mocked data obtained from these processes are not associated with any real person and are solely used for demonstration purposes. The associated rows in the sequence-based allele table also moved with their swapped genotypes.

## Searching module methods

The STRategy provides two sorts of searches: overview search and profile search. The overview search utilizes the set of genotypes and loci to query and return the number of matched samples for public users and the sample IDs, countries, and provinces for laboratory users. The profile search requires a specific set of genotypes and loci and provides a correlation with statistical data. For the overview search, the system will search for samples that consist of a set of loci and genotypes specified by the user. For example, the user defines the loci and genotypes as D12S391:19,25 and TPOX:8,8. The algorithm then looks for samples that must include these loci and genotypes.

On the other hand, the profile search behaves similarly to OmniPop200.1. The user must first pick the core loci as the reference. After that, the user must specify the search input with minimal loci and genotypes of the sample according to the selected core loci. For example, if the user chooses core loci as European, the minimal loci are FGA, TH01, VWA, D1S1656, D2S441, D3S1358, D8S1179, D10S1248, D12S391, D18S51, D21S11, and D22S1045. With the genotypes of the core loci as input, the STRategy will use Eqs (1) and (2) [12] to calculate the homozygote and heterozygote frequencies. Then, the algorithm will multiply the reciprocal genotype frequencies from the same country and rank these values across the countries. The lowest value means the input profile is most common in that country.

$$F(x)=p^{2}+p\cdot \left(1-p\right)\cdot \theta$$ (1)

Where *p* is the allele frequency value

*θ* is an empirically determined measure of population subdivision

F(x)=2⋅p⋅q (2)

Where *p* is the first allele frequency value

*q* is the second allele frequency value

### The STR pattern alignment settings

We obtained the reference STR repeat motifs built-in with STRategy from STRidER [13] and added a flanking sequence for some loci. However, the STRategy also allows administrators to define each locus’s reference STR repeat motifs. This setting is in an Excel file; an administrator must upload it. The file includes the locus name, the reference STR repeat motifs formatted according to the conventions used in [14], the allele, and the orientation columns (S2 Fig). The allele column with the “Default” value indicates that the pattern applies to all alleles in this locus. If an administrator wants the system to analyze an allele with a microvariant pattern, the administrator must add this microvariant explicitly into the system. For example, S2 Fig shows the additional allele 10.3 of locus D2S441 explicitly added by the administrator, which has a pattern different from other alleles within the same locus. Finally, the orientation column indicates the order of the samples’ sequence. The sequences in each locus of a sample details report file can be either forwarded or reversed. The STRategy states that the reference STR repeat motifs must always be the forward strand (the sequence in the 5’ to 3’ direction). Therefore, the administrator can set reverse or forward in the orientation column to handle the strand direction of samples’ sequences. The reverse orientation tells the system to reverse complement the reference STR repeat motifs before analyzing pattern alignment. For example, the reference STR repeat motifs of CSF1PO (row 3 in S2 Fig) is [ATCT]n in the forward strand. However, the administrator notices sequences in sample details report files are in the reverse strand direction. Hence, the administrator must set the “Reverse” value in the orientation column. Then, based on these STR repeat motifs, the administrator can generate pattern alignment using a tool on the administrator’s menu. This tool compares the reference STR repeat motifs of the locus to the DNA sequence within each sample locus. It detects STR repeat motifs and any insertion or deletion with a length less than the length of the reference motif.

### User management

The STRategy system provides three distinct user roles: public users, laboratory users, and administrators. Each role has its own set of permissions for reading and manipulating data within the system. For example, the system does not require public users to log in. In contrast, laboratory users and administrators must log in to access system functionalities related to data privacy. As a result, laboratory users and administrators are assigned one of four statuses (Table 1): NOT_ACCEPTED, ACCEPTED, BLOCKED, or DELETED. When new users register to the system, they are initially assigned the NOT_ACCEPTED status and the laboratory user role. However, they can only access the system once an administrator approves their account. After administrator approval, their status would change to ACCEPTED, granting them access to the system with the appropriate permissions for their designated role.

**Table 1:** Description of account statuses.

Status	Description
NOT_ACCEPTED	Assigned to new users who have registered to the system but have yet to be approved by an administrator. These users can only access the system once their account is approved.
ACCEPTED	Assigned to users who have been approved by an administrator and can access the system with the appropriate permissions for their role.
BLOCKED	Assigned to users who are no longer in the laboratory or have had access to the system revoked by an administrator due to other issues. These users can no longer access the system.
DELETED	Assigned to users whose accounts have been permanently deleted by an administrator. These users can no longer access the system, and their data is typically removed. Note that deletion is a more severe action than blocking, as blocked users may be able to have their access reinstated, while deleted users cannot. Therefore, administrators should exercise caution when choosing to delete a user account.

Public users have limited permissions within the system, allowing them to view only the aggregated data. In contrast, laboratory users have broader access and can execute more sophisticated operations, such as data manipulation and analysis. If an administrator chooses to BLOCK or DELETE a user’s account, the user will lose all access to the system, irrespective of their prior status or role.

Overall, we designed the STRategy system to deliver different levels of access and control based on user roles and statuses. By distinguishing between user categories and their respective permissions, the system ensures that data is available only to those with appropriate authorization while enabling administrators to manage user accounts effectively.

## Results

### Searching modules

**Fig 2:**
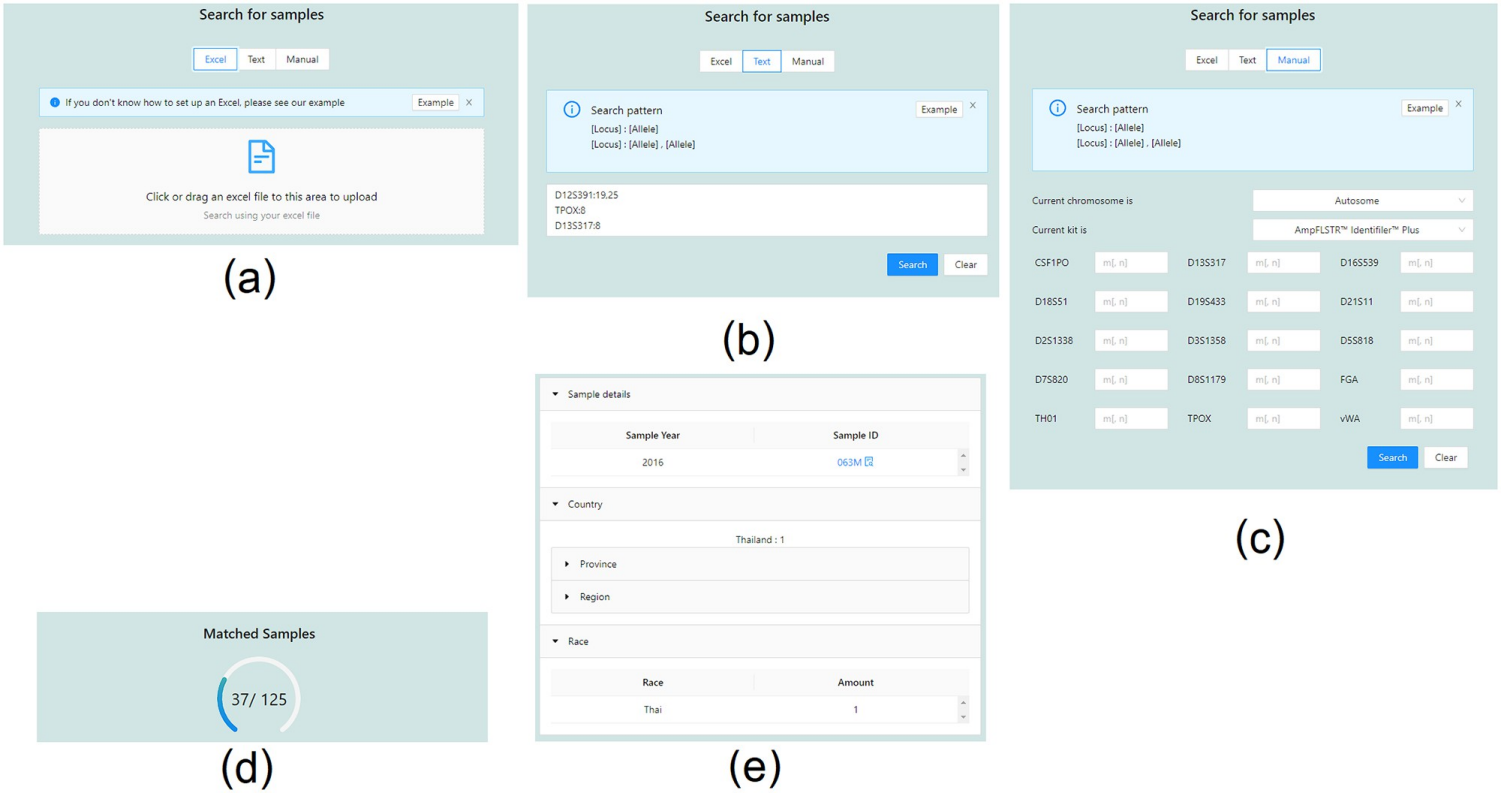
Overview search options. (a) search by ForenSeq sample detail report file of a sample (b) search by any loci and genotypes (c) search by any loci and genotypes of a specific test kit (d) search results for public users (e) additional search results for laboratory users.

The STRategy provides three search options for overview search. The first option allows users to upload a sample file as input (Fig 2a), which is then searched against all available samples within the system. The second and third options are equivalent in using a set of genotypes and loci as the input. However, the second option (Fig 2b) allows an independent set of loci, while the third option (Fig 2c) allows only the loci corresponding to the selected kit. All options use the input genotypes and loci to search against all samples within the system and return only the number of matched samples for public users (Fig 2d). Laboratory users will also get additional information, including the number of matched samples grouped by geographical location, sample IDs, nations, and provinces (Fig 2e).

**Fig 3:**
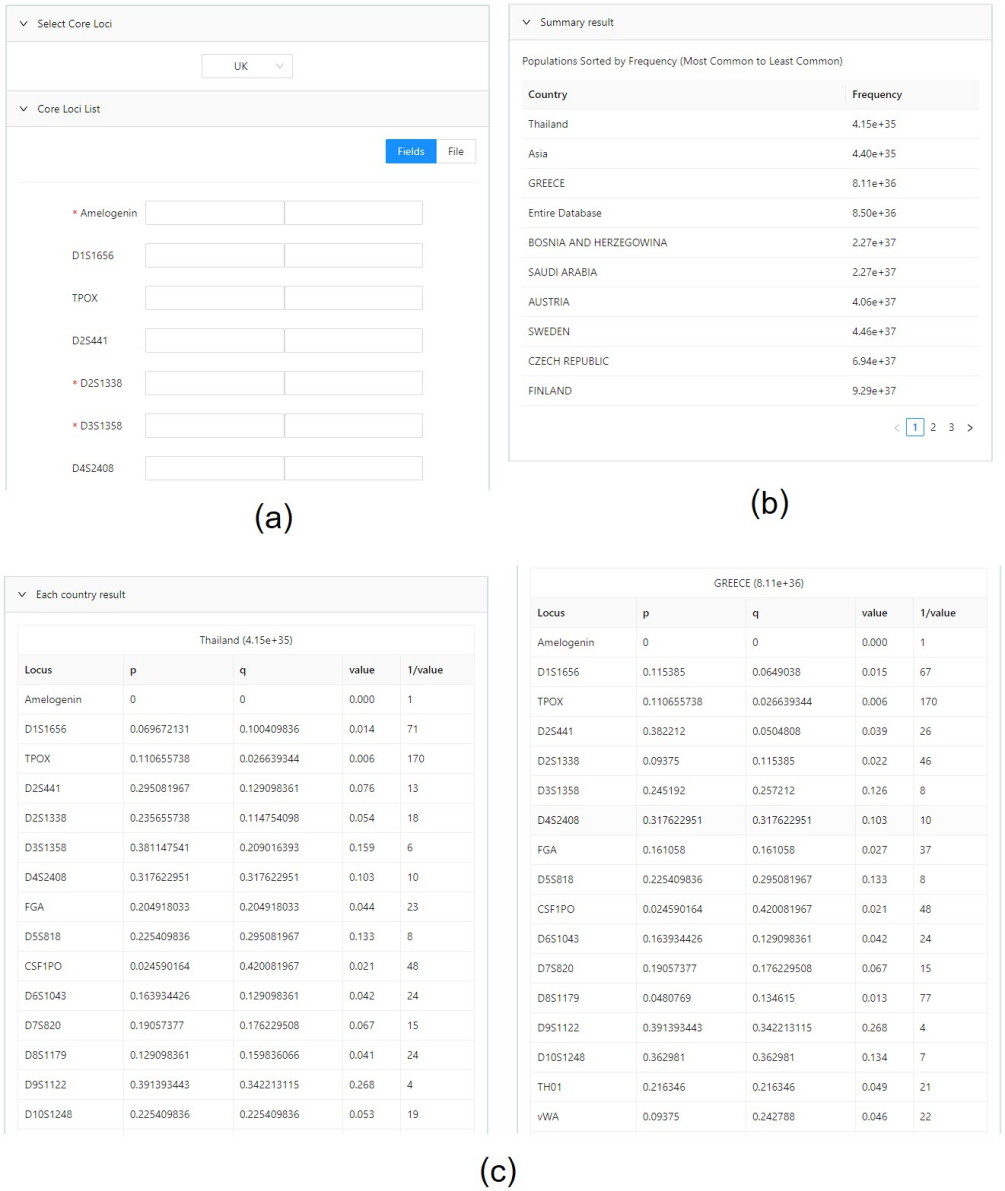
Profile search. (a) input form (b) countries with most common genotype frequencies (c) detailed genotype frequencies of each country and locus.

The profile search (Fig 3) lists the required “core STR loci.” The system obtained the core STR loci used for human identification from STRBase [15] and length-based allele frequency data from STRidER [16, 17] as defaults for the correlation calculation. After selecting core loci, the user must provide genotypes for each required locus. Users can input genotypes using one of two options: manually entering genotypes in the Fields tab or uploading an input file in the File tab (Fig 3a). After submitting the genotypes in the input form, the system displays the results with a table of the countries sorted by the most common genotype frequencies (Fig 3b) and detailed tables of the length-based allele frequencies calculated from each country (Fig 3c). The administrator can customize the core STR loci and the length-based allele frequency data (See section system management by administrators).

### Statistics

**Fig 4:**
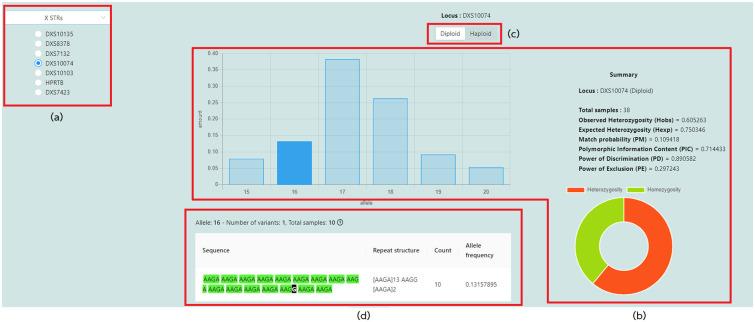
Genetic variation page. (a) list of selectable chromosome and loci (b) allele frequencies and common statistical values for assessing genetic variation of the samples (c) diploid or haploid toggle (d) genetic variation of the selected locus DXS10074 with allele 16.

**Fig 5:**
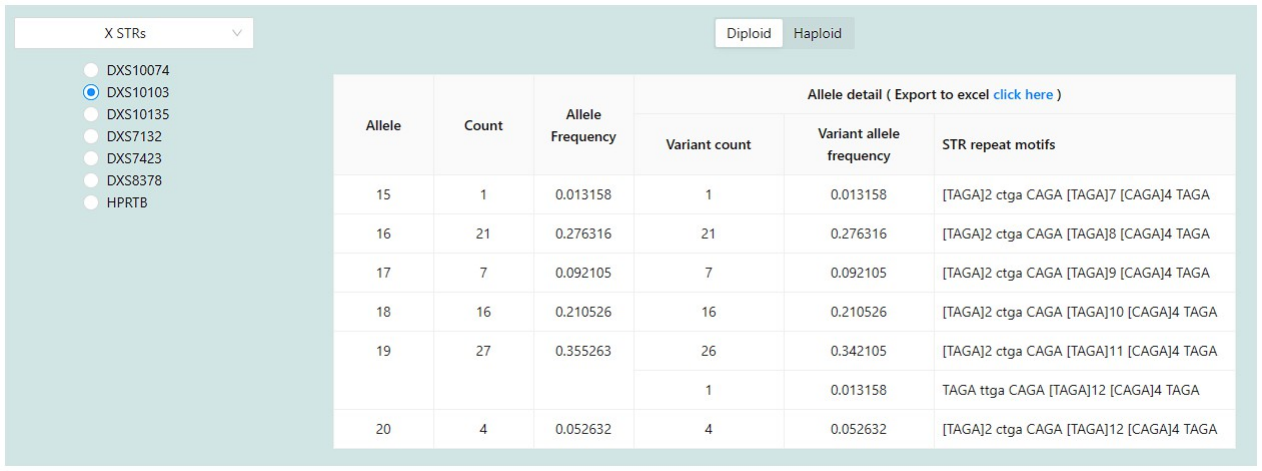
Allele details page.

Users can explore the statistical data analyzed by the system via different web pages. The genetic variation (Fig 4) and allele details (Fig 5) pages display the allele frequencies for each locus. On the left side of the genetic variation page (Fig 4a), users can select an autosomal, X, or Y chromosome and a locus from the available list. Once a chromosome and a locus have been selected, the system will display a bar graph of length-based allele frequencies and statistical values on the right-hand side (Fig 4b). Alleles in that locus can be divided into three cases. First, the locus comprises haploid genotypes only (S3 Fig). Therefore, the system will display the Probability of Matching (PM), Polymorphic Information Content (PIC) [18], and Power of Discrimination (PD) values. Second, the system will display the Observed Heterozygosity (Hobs), Expected Heterozygosity (Hexp), Probability of Matching, Polymorphic Information Content, Power of Discrimination, and Power of Exclusion (PE) if the locus contains diploid genotypes only (S4 Fig). Third, if the locus contains both haploid and diploid genotypes, for example, a locus of the X chromosome, the system will allow the user to toggle between the Diploid and Haploid (Fig 4c). Fig 4d displays a table containing all sequence-based allele variations when the user clicks an allele bar from the bar chart in Fig 4b. Furthermore, the allele details page (Fig 5) presents a comprehensive summary of all STR variations and their corresponding allele frequencies for each variation.

**Fig 6:**
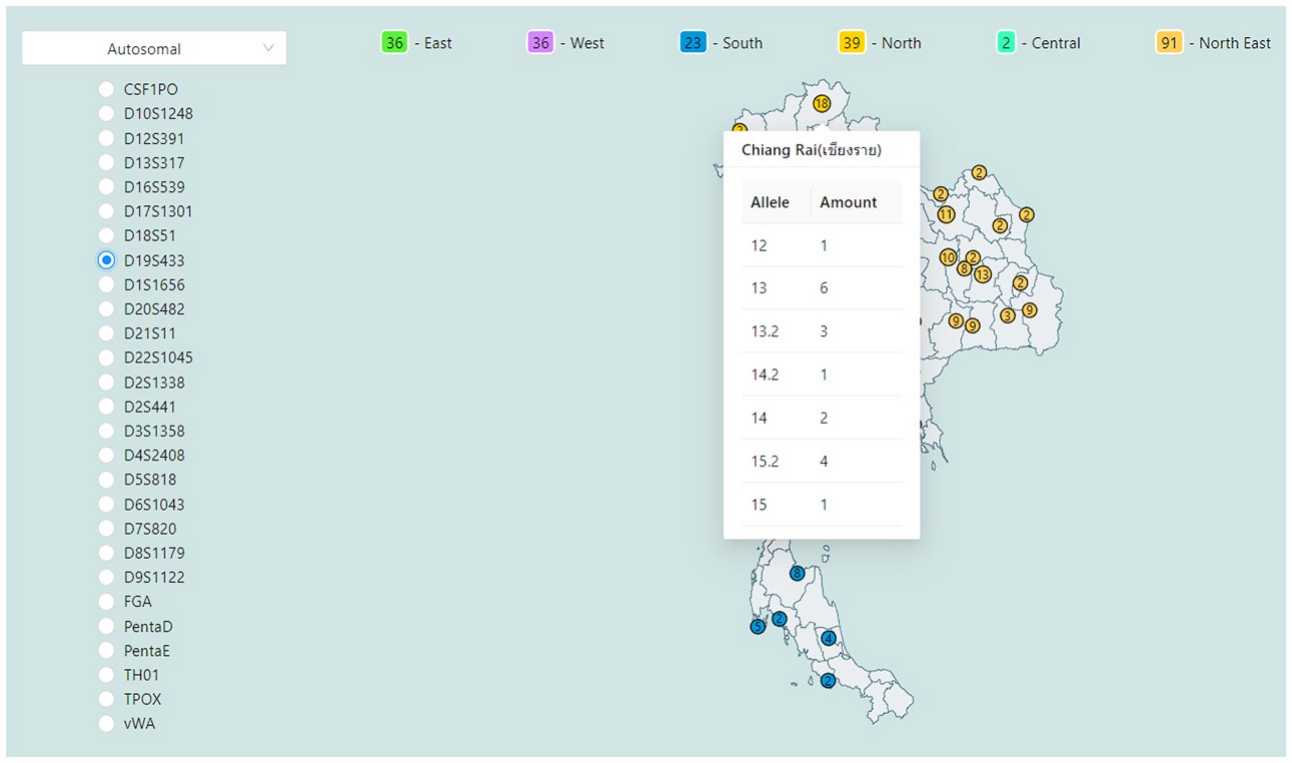
Allele distribution of a selected locus by geographic location. (Map source: https://geodata.ucdavis.edu/gadm/gadm4.1/shp/gadm41_THA_shp.zip, license: https://gadm.org/license.html).

Allele distribution by geographic page shows the map of samples with regional data. Each circle on the map represents the number of samples. Hovering the cursor over a circle will display all length-based alleles found within the province (Fig 6). Additionally, the user may mouse over a square to see the number of samples collected from each region. Furthermore, the administrator can configure the map on this page to any country (See section system management by administrators).

The iSNP statistic summary (S5 Fig) page displays data aggregated by SNP locus and allows for searching by locus name. The bar graph illustrates the summary of the altered nucleotide pairs of all samples within the database. The mouse hovering over each bar shows the proportion of each SNP variation.

The allele frequency comparison page (S6 Fig) provides a comparative view of length-based allele frequency from all samples within the STRategy’s local database samples and international populations obtained from STRidER [16]. Users can explore the allele frequency of a specific locus by selecting from the drop-down list. The system will then generate a two-dimensional grid in which each row represents the allele, and each column indicates the country. Each cell represents the allele frequency by color and shows the value when mouse over.

### The STR pattern alignments

**Fig 7:**
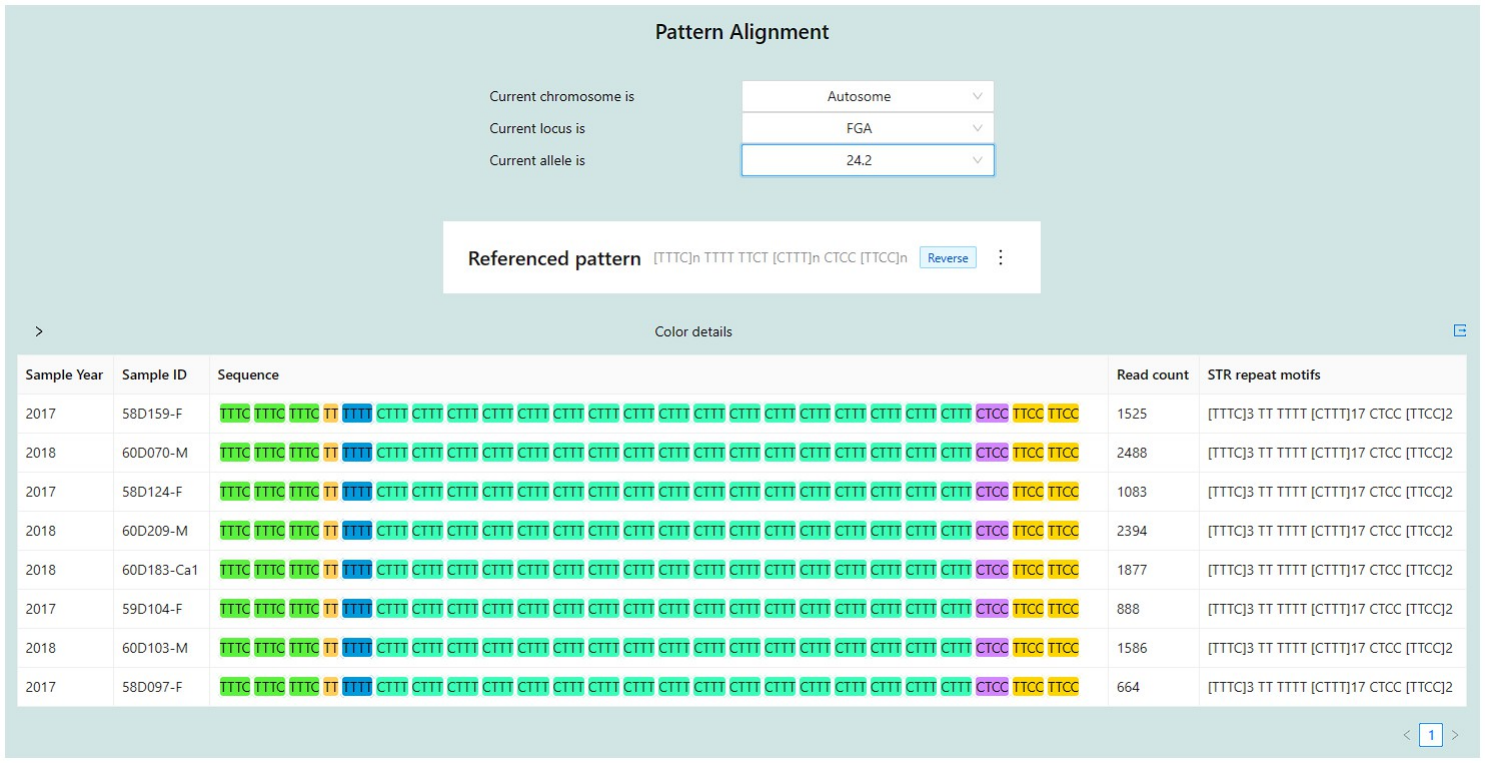
Pattern alignment page.

The STR pattern alignment page allows users to browse through chromosomes, loci, and alleles. After selection, it will display tabular data in five columns: Sample Year, Sample ID, Sequence, Read count, and STR repeat motifs, where the Sequence column contains the sample nucleotide sequence with each STR motif highlighted individually (Fig 7), and the STR repeat motifs column includes a summary of the sequence’s STR. This data is not accessible to public users. Only laboratory users and administrators can access this page. Fig 7 shows the result of the pattern alignment of all samples in the database with a specific locus FGA and allele 24.2. The system displays the reference STR repeat motifs on the top of the pattern alignment table, telling the user that its pattern within the sequence is reverse or forward as pre-specified by the administrator in S2 Fig. When the administrator uploads a sample details report file to the system where sequences in the CSF1PO locus are reversed, the administrator must specify the pattern of this locus as reverse in the orientation column, and the STRategy will display sequences on this page as the reverse. On the contrary, the sequences from the locus D10S1248 in the sample details report file are forwarded; hence, the orientation column of this locus must be pre-specified as forward. So, the system will display them as forward. However, users can view the forward or reverse directions by toggling the referenced pattern tag or kebab menu. For example, Fig 7 shows that the reference STR repeat motifs are reversed. It means the original sequences of this locus within the sample details report file are in the reverse direction. On the other hand, if users toggle the referenced pattern, all sequences will be forward, as shown in S7 Fig.

Laboratory users can also export the pattern alignment results in Excel formats, i.e., XLS and XLSX extensions, via the export page (S8 Fig). S9 Fig shows an example result of the exported alignment patterns with five columns: Sample ID, Sample Year, Allele, Repeat Structure, and Sequence.

### Data management by laboratory users

**Fig 8:**
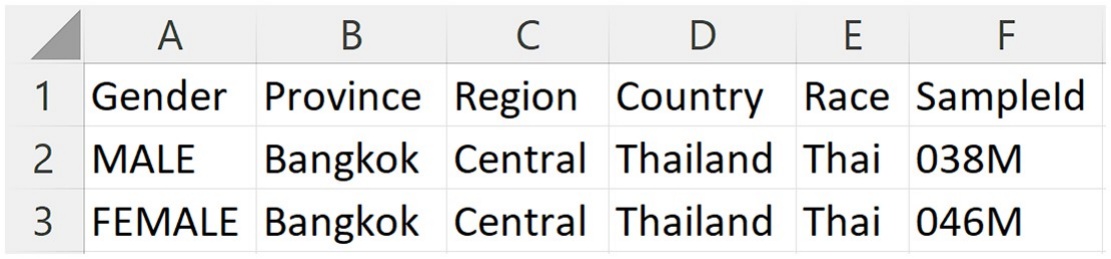
Personal information in excel file format.

Laboratory users can access all public functions and upload STR ForenSeq sample detail report files to the system via the upload STR data page (S10 Fig). The system assumes that the laboratory users have validated the files following the ForenSeq manual and their data validation protocol. When the laboratory user uploads the file, the system checks the sample ID to see whether it duplicates a sample ID already in the system. If this is the case, the system offers the user the choice of canceling or overwriting the sample data (S11 Fig). After uploading sample details report files, laboratory users can also upload the corresponding personal data file (Fig 8) for each sample or as a batch of persons via the upload personal data page (S12 Fig). The system links these personal data based on the sample ID. Laboratory users can edit or delete personal data via the manage personal data page (S13 Fig). The system will then look for values in the Province, Region, Country, and Race columns and associate them with the sample ID. An administrator must enter these provinces, regions, countries, and races into the system before a laboratory user uploads this personal data file.

### System management by administrators

Administrators can perform the same functions as public users and laboratory users. Additionally, they can customize the system by, for example, changing the map and managing users (S14 Fig). The STRategy system offers the administrator’s default username and password for the initial login, whose password can be changed later.

Administrators may use any country’s map for map representation (S15 Fig) for visualizing allele distribution by geographic location (Fig 6). The map visualization was created using React Simple Maps [19], which supports data in TopoJson [20] or GeoJson [21] formats. To utilize the map within this system, an administrator can obtain an example of a TopoJson world map file from [22] and upload it to the system through this feature. Moreover, the administrator can modify the center of the map depending on the latitude and longitude grid system and the map amplification scale to accommodate the data in their local database. However, a large map file can significantly impact the system’s performance and slow down the display speed. In such cases, users can consider generating a customized TopoJson file from shapefile data using the conversion guide available in [23]. In addition, the administrator may download the shapefile data for all countries from [24]. These sources allow the administrator to get a new map that meets their needs while avoiding the negative impact of large map files on the system’s performance.

The system also provides web pages for setting the profile search. The first page is to upload the core loci to the system or replace the existing ones with new ones (S16 Fig). This file’s format is an Excel file consisting of two columns: locus and country (S17 Fig). The default core loci within the file will also appear as part of other countries’ core loci. For example, the default core loci are FGA, TH01, TPOX, and vWA, and a country ABC’s core loci are only FGA and TPOX. When displayed on the profile search, users will see all the default core loci: FGA, TH01, TPOX, and vWA, where FGA and TPOX will be marked as required. The second page is to upload length-based allele frequencies (S18 Fig). The STRategy has default length-based allele frequency data from STRidER [16]. However, the administrators can replace default data by uploading their Excel files. There are two upload options to replace length-based allele frequencies in the database. First, upload allele frequencies of all countries simultaneously with an Excel file, as shown in S19 Fig. This Excel file contains length-based allele frequency tables; each table represents the allele frequencies of a locus. All tables have a locus name on the top of the tables. The second option is uploading allele frequencies of all loci by country (S20 Fig). This Excel file does not have a country name, but the administrator must choose the country before uploading the file (S21 Fig). The last page (S22 Fig) is to set up the theta constant of Eq (2) (homozygous genotype equation).

Additionally, administrators can add new loci and kits (S23 Fig) to the system. They can also add and modify countries and create, update, and remove races (S24 Fig), regions, and provinces (S25 Fig) associated with each country (S26 Fig). Then, the geographic page uses this information to display the map correlated with the uploaded samples’ personal data (Fig 8). Finally, they may submit reference STR repeat motifs and regenerate repeat motifs of the samples for the STR pattern alignments. When laboratory users submit new samples to the system, or administrators upload new reference STR repeat motifs for pattern alignment, the system will inform administrators to conduct the regeneration of new STR pattern alignments (S27 Fig).

### Implementations

The STRategy frontend was implemented based on React library [25] version 16.3. React renders the virtual Document Object Model (DOM), which works fast as it only changes individual DOM elements instead of reloading the complete DOM every time. Also, it provides reusable components making it easier for the developer to develop the system. We implemented the STRategy backend using Spring Boot [26], an open-source micro-framework built on top of the Spring framework, as the forensic data is sensitive to data privacy. The Spring framework provides industry-standard security schemes and delivers a trustworthy solution. The system uses MySQL database version 5.7 [27] to store all the data.

### Validations

We calculated the allele frequencies and statistical values using 125 mocked samples imported into the STRategy as the showcase and compared them to the results from STR Analysis for Forensic (STRAF). All five comparison results of allele frequencies and statistical values: autosomal (diploid), x (diploid), x (haploid), y (diploid), and y (haploid), arranged as five folders were compressed into the S1 File (See Additional data section). Each folder has three sub-folders inside. The first sub-folder contains allele frequency results. This folder has three files: results from STRAF (S28 Fig), results from STRategy (S29 Fig), and compared results from both (S30 Fig). The second sub-folder contains statistical results with three files: results from STRAF (S31 Fig), results from STRategy (S32 Fig), and compared results from both (S33 Fig). The last sub-folder contains raw data files uploaded to STRAF. We validated allele frequencies and statistical values to 5 decimal places for all loci listed in S1 Table. For diploids, we compared STRategy and STRAF by five values: Polymorphic Information Content, Probability of Matching, Power of Discrimination, Heterozygosity, and Power of Exclusion. STRategy and STRAF, respectively, reference the formula from [28, 29] to calculate Gene Diversity (GD). So, we did not compare GD results in this validation. For haploids, we measured STRAF and STRategy by three values: Polymorphic Information Content, Probability of Matching, and Power of Discrimination. The validation results show that all values calculated by STRategy are consistent with all values computed by STRAF.

We validated the pattern alignment results from STRategy by comparing them with those from STRait Razor 3.0. First, we randomly selected 5 out of 125 samples within the STRategy. After that, we used STRait Razor to get haplotypes of short tandem repeats from the FASTQ files of those five samples and imported the result files from STRait Razor into the Excel-based workbook of STRait Razor 3.0 [30] from Strait Razor’s GitHub [31]. This workbook will display the sequences and repeat structures for each sample. We then compared the alleles’ repeat structure of every locus reported by STRait Razor (S2 Table) with the repeat structures of our pattern alignment results. Finally, we saved the comparison results as Excel files and compressed them S2 File (See Additional data section). We also validated all other alleles (S3 Table) not listed in the first five samples’ loci with all possible cases of insertion-deletion (indel), which can cause incorrect pattern alignment analysis by randomly choosing ten more samples to cover these cases. In contrast to the five previously described samples, we evaluated these ten samples for only specific loci and alleles with indels. As STRait Razor did not report the repeat structure of some alleles in some loci, for example, allele 15.3 of locus D8S1179, we omitted the comparison of those alleles. Nevertheless, the validation results show that all pattern alignments’ repeat structures generated by STRategy agreed with all repeat structures identified by the Excel-based workbook of STRait Razor 3.0. We focused on validating only the repeat regions since flanking regions may vary across software and could lead to inconsistencies in the validation process.

## Discussions

This paper presents the STRategy, an end-to-end support system for collecting and analyzing next-generation sequencing data of short tandem repeats for forensic science. The STRategy allows laboratory users to manage and store STR data efficiently and organizationally. In addition, it provides automated analysis capabilities, which enable users to interpret and visualize their results quickly.

Without STRategy, if a laboratory user has ForenSeq sample detail reports and wants to calculate forensic parameters, the user could use STRAF. However, one drawback of using STRAF is that the user needs to convert all of the sample detail reports to the format specified by STRAF before uploading them into its system for analysis. This conversion step can take time and effort, especially when dealing with extensive data. Furthermore, suppose a laboratory user has new ForenSeq sample detail reports and wants to recalculate the forensic parameters. In that case, the user must repeat the conversion process for all reports, including previous ones. In contrast, STRategy simplifies this process by allowing direct upload of ForenSeq sample detail reports without any conversion. Once uploaded to STRategy, the system automatically calculates forensic parameters and displays them via various analysis results pages, such as the genetic variation and allele details pages. Additionally, STRategy automatically recalculates the parameters when new ForenSeq sample detail reports are uploaded, streamlining the data analysis process and saving time.

In addition to its ability to manage and store STR data efficiently, STRategy offers pattern alignment analysis with its identified repeat structures agreed with STRait Razor. However, STRait Razor requires users to start from the FASTQ files of a sample and go through several processing steps, such as identifying alleles from reads in FASTQ files, inputting the result file into the Excel-based workbook, and viewing the result of repeat structures. In contrast, with STRategy, the process is simplified as laboratory users can directly upload the ForenSeq sample detail reports and run the pattern alignment analysis through the system. This flow eliminates the need for extra steps and makes STRategy a more user-friendly option for identifying STR repeat motifs and collecting the report files into the database simultaneously. In addition, this feature helps align and visualize sequences of STR repeat motifs of the same locus from all samples automatically, which can result in discovering SNPs and indels within the STRs of the same locus among the samples.

Another strength of the STRategy is its use of Docker, which allows users to run the software in a containerized environment without needing specific hardware or software configurations. Users can download, install, and run a copy of STRtegy on their local computer or a computing instance on the cloud using the Docker engine without worrying about compatibility issues. In addition, the STRategy includes Role-based Access Control (RBAC), which enables users to set different access levels and permissions for different individuals or groups. This access control ensures that sensitive data is protected and that only authorized personnel can access and modify it.

One limitation of the STRategy system is that its current version relies on only a specific sequencing technology: ForenSeq Signature Prep of Illumina platform. This dependence may limit its application to laboratories that do not have access to this technology or may require additional validation studies for other sequencing platforms. However, we acknowledge this limitation and highlight the system’s ongoing development to expand its compatibility with other sequencing platforms.

## Conclusions

The STRategy presents a user-friendly and efficient solution for collecting and analyzing next-generation sequencing STR data from sample detail reports in forensic science. The system delivers essential data management capabilities and several visualizations allowing users to explore the analyzed data interactively. With the added convenience of essential analysis tools, such as automated forensic parameter calculation, STR pattern alignment, and profile search, the STRategy reduces the time and effort required to manage and analyze STR data using multiple tools. Moreover, the STRategy’s flexible customization features allow forensic laboratories or organizations to tailor the system to their specific needs, such as changing the map to different countries, adjusting the pattern alignment settings, or adding a new test kit with its corresponding loci. Moreover, the system’s use of Docker for easy deployment on various operating systems, e.g., Windows, Linux, MacOS, and RBAC for data protection, adds to its user-friendliness and versatility. We plan to include STR files from other NGS platforms to expand the system’s capabilities. Overall, the STRategy presents a helpful solution for managing and analyzing STR data from NGS in forensic science, with implications for streamlining and optimizing current practice.

## Supporting information

S1 Fig SNP data sheet from next-generation sequencing (ForenSeq). (a) SNP genotype and (b) SNP allele read coverage information.
(TIF)

S2 Fig Example of reference STR repeat motifs in excel file format. (TIF)

S3 Fig Genetic variation page with only haploid genotypes under the statistics section. (TIF)

S4 Fig Genetic variation page with only diploid genotypes. (TIF)

S5 Fig iSNP statistical summary page under the statistics section. (TIF)

S6 Fig STRategy and STRidER allele frequency comparison page of locus D3S1358 under the statistics section. (a) allele frequency calculated from all samples within the STRategy database (b) allele frequency of various countries obtained from STRidER.
(TIF)

S7 Fig Pattern alignment page with forward reference pattern under the laboratory user section. (TIF)

S8 Fig Pattern alignment export page under the laboratory user section. (TIF)

S9 Fig An exported Excel file of the pattern alignment result. (TIF)

S10 Fig Upload STR data page under the laboratory user section. (TIF)

S11 Fig Laboratory user uploads duplicated data to the system. (TIF)

S12 Fig Upload personal data page under the laboratory user section. (TIF)

S13 Fig Manage personal data page under the laboratory user section. (TIF)

S14 Fig Manage user page under the administrator section. (TIF)

S15 Fig Manage map admin page under the administrator section. (a) Preview page (b) Configuration panel.
(TIF)

S16 Fig Web page for managing core loci under the administrator section. (TIF)

S17 Fig An example of Excel file for core loci upload. (TIF)

S18 Fig Web page for uploading allele frequency of all countries under the administrator section. (TIF)

S19 Fig Length-based allele frequencies of all countries. (TIF)

S20 Fig Length-based allele frequencies of a country. (TIF)

S21 Fig Web page for uploading allele frequency by a country. (TIF)

S22 Fig Web page for managing theta constant under the administrator section. (TIF)

S23 Fig Web page for managing kits and loci under the administrator section. (TIF)

S24 Fig Web page for managing races under the administrator section. (TIF)

S25 Fig Web page for managing provinces under the administrator section. (TIF)

S26 Fig Web page for managing countries under the administrator section. (TIF)

S27 Fig Notification to administrators for the management of pattern alignment under the administrator section. (TIF)

S28 Fig Allele frequencies result file from STRAF (X diploid). (TIF)

S29 Fig Allele frequencies result file from STRategy (X diploid). (TIF)

S30 Fig Allele frequencies comparison result file from both STRAF and STRategy (X diploid). (TIF)

S31 Fig Statistical values result file from STRAF (X diploid). (TIF)

S32 Fig Statistical values result file from STRategy (X diploid). (TIF)

S33 Fig Statistical values comparison result file from both STRAF and STRategy (X diploid). (TIF)

S1 File Length-based allele verification results. (ZIP)

S2 File Pattern alignment verification results. (ZIP)

S1 Table Loci list with allele frequencies and statistical values validated with STRAF. (XLSX)

S2 Table Loci list in with repeat structures validated with STRait Razor. (XLSX)

S3 Table Additional alleles with repeat structures validated with STRait Razor. (XLSX)
