# Supplementary figures and images for: STRategy: A support system for collecting and analyzing next-generation sequencing data of short tandem repeats for forensic science
Source: PLoS One. 2023 Jul 17;18(7):e0282551. doi: 10.1371/journal.pone.0282551 (PMC10351723; doi:10.1371/journal.pone.0282551)

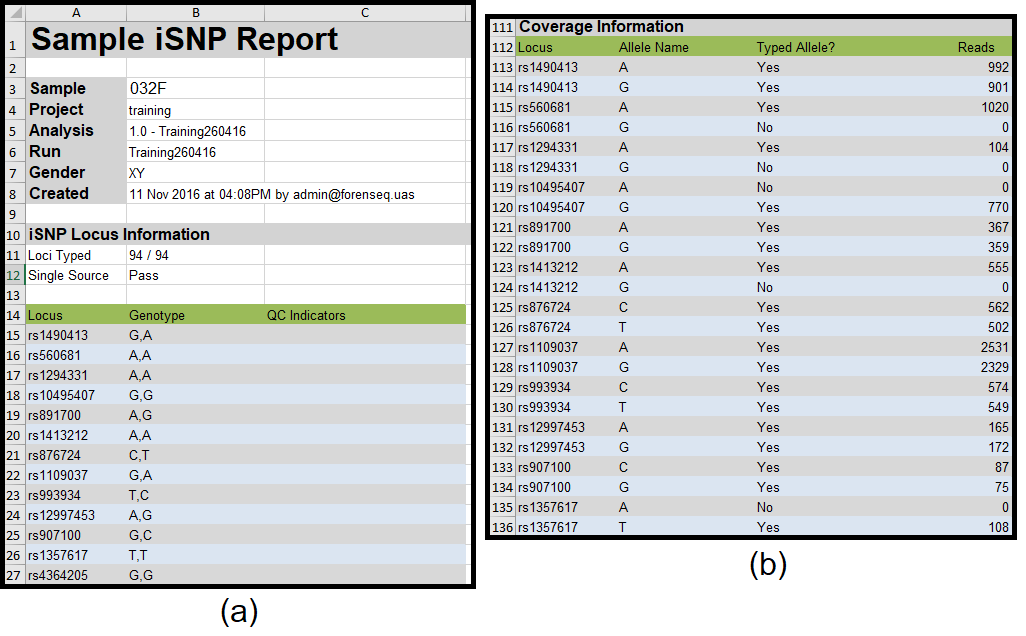

Supplement: S1 Fig — (a) SNP genotype and (b) SNP allele read coverage information. (TIF) [file pone.0282551.s001.tif]

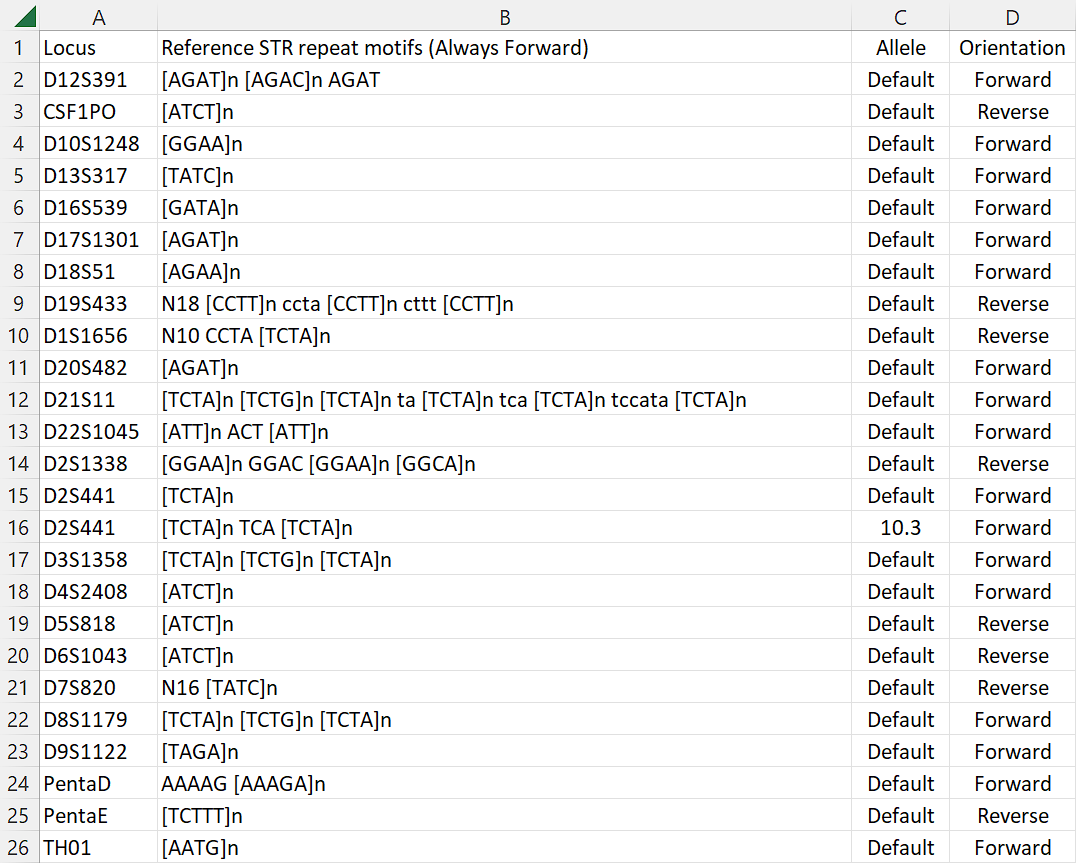

Supplement: S2 Fig — (TIF) [file pone.0282551.s002.tif]

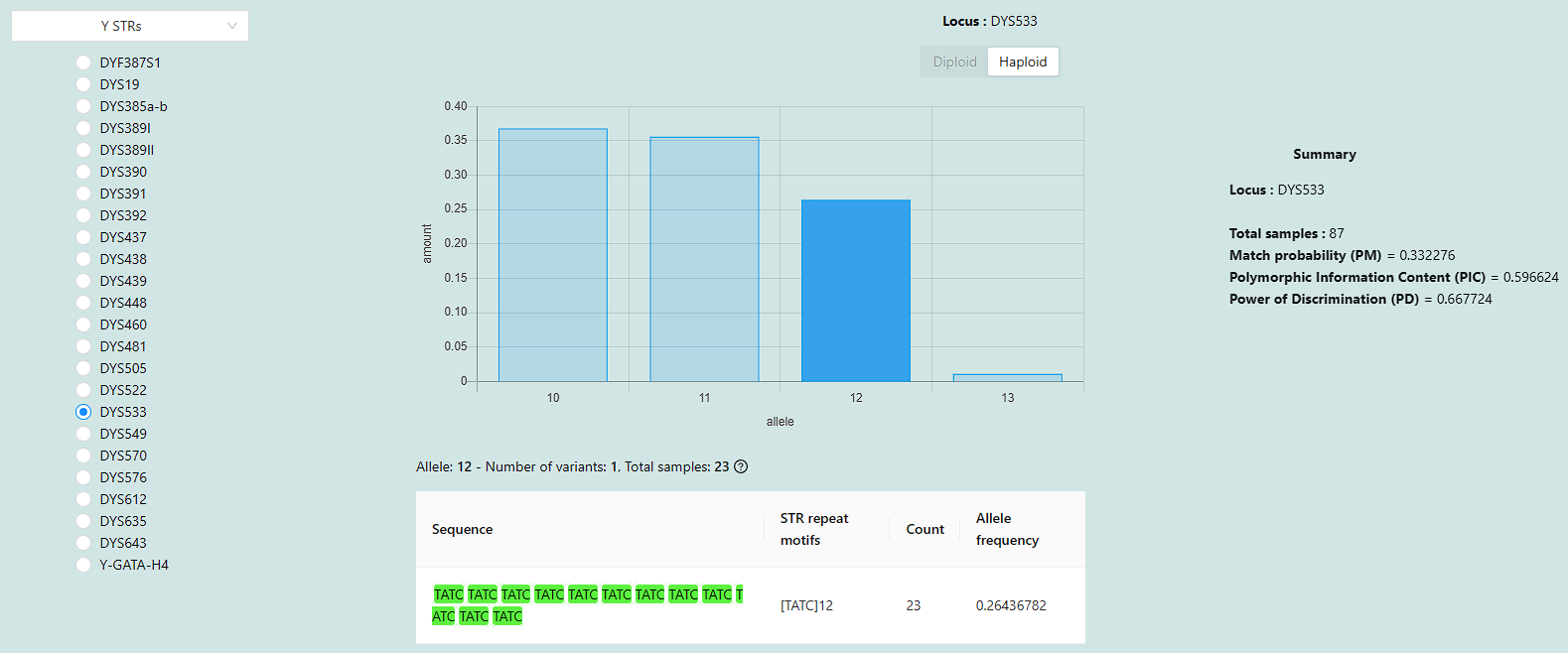

Supplement: S3 Fig — (TIF) [file pone.0282551.s003.tif]

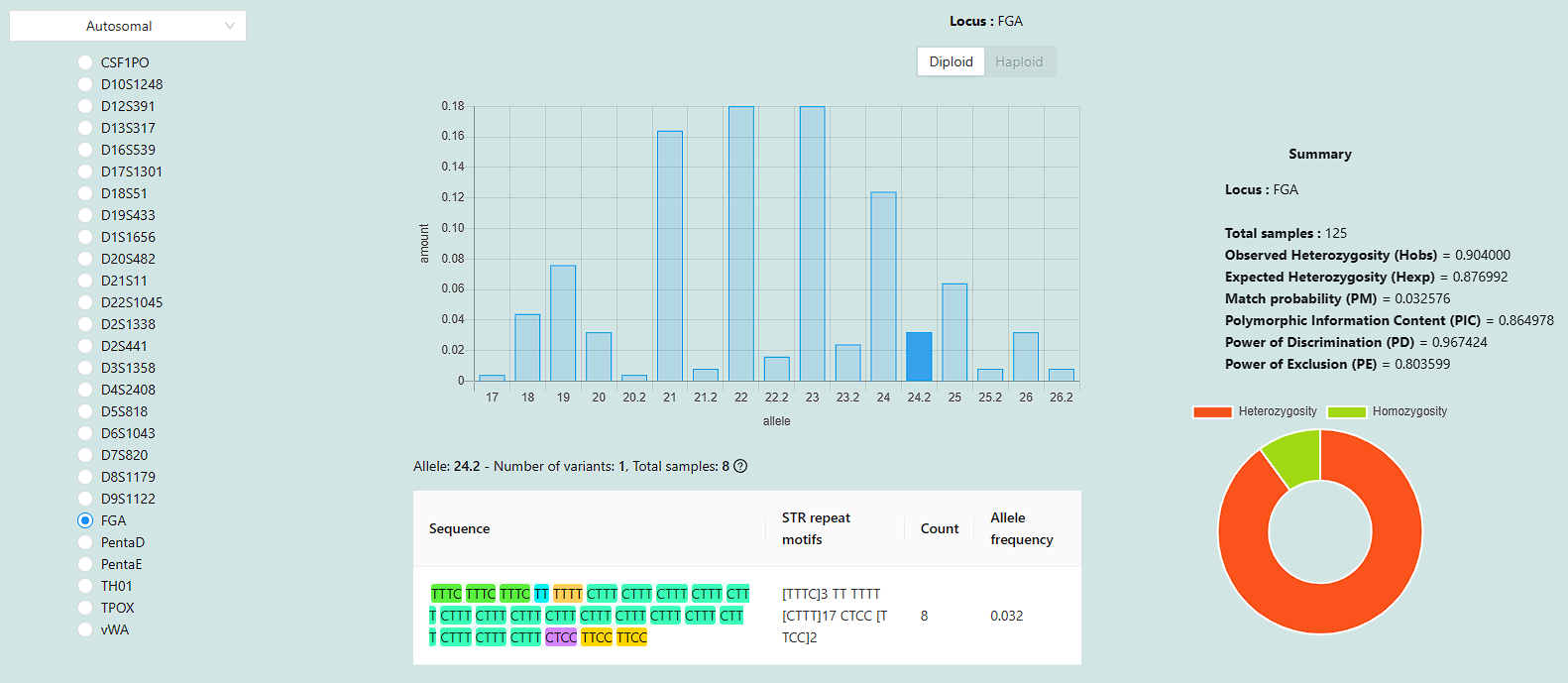

Supplement: S4 Fig — (TIF) [file pone.0282551.s004.tif]

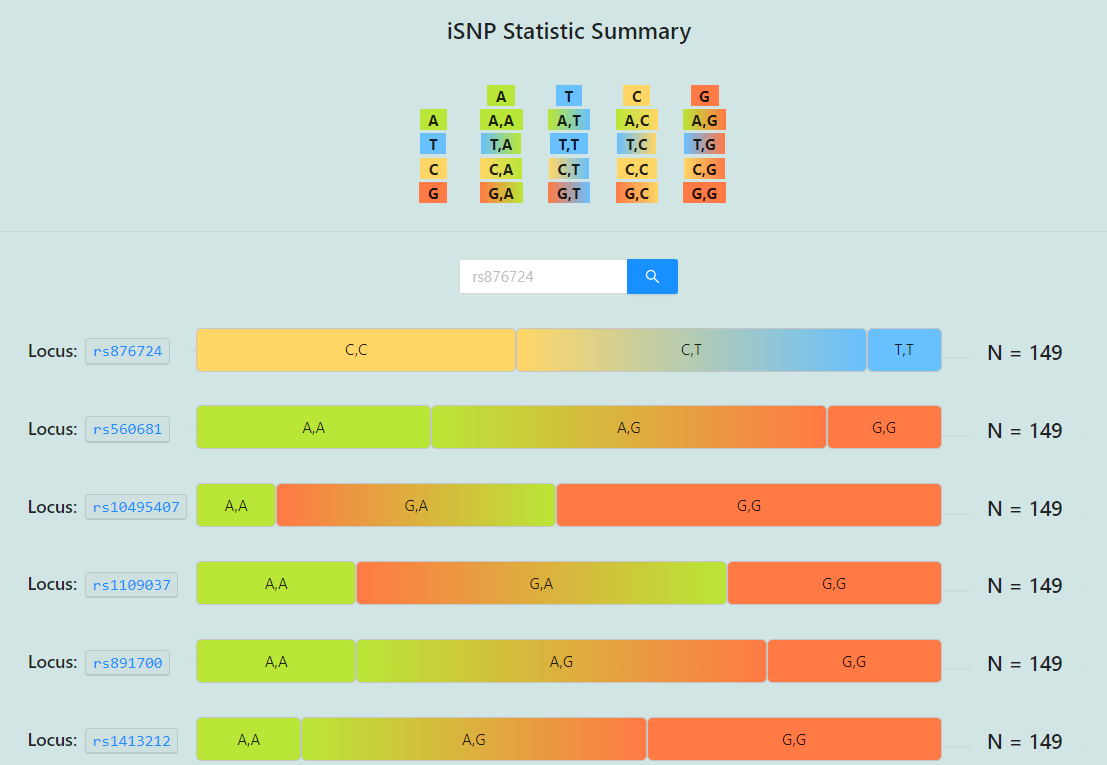

Supplement: S5 Fig — (TIF) [file pone.0282551.s005.tif]

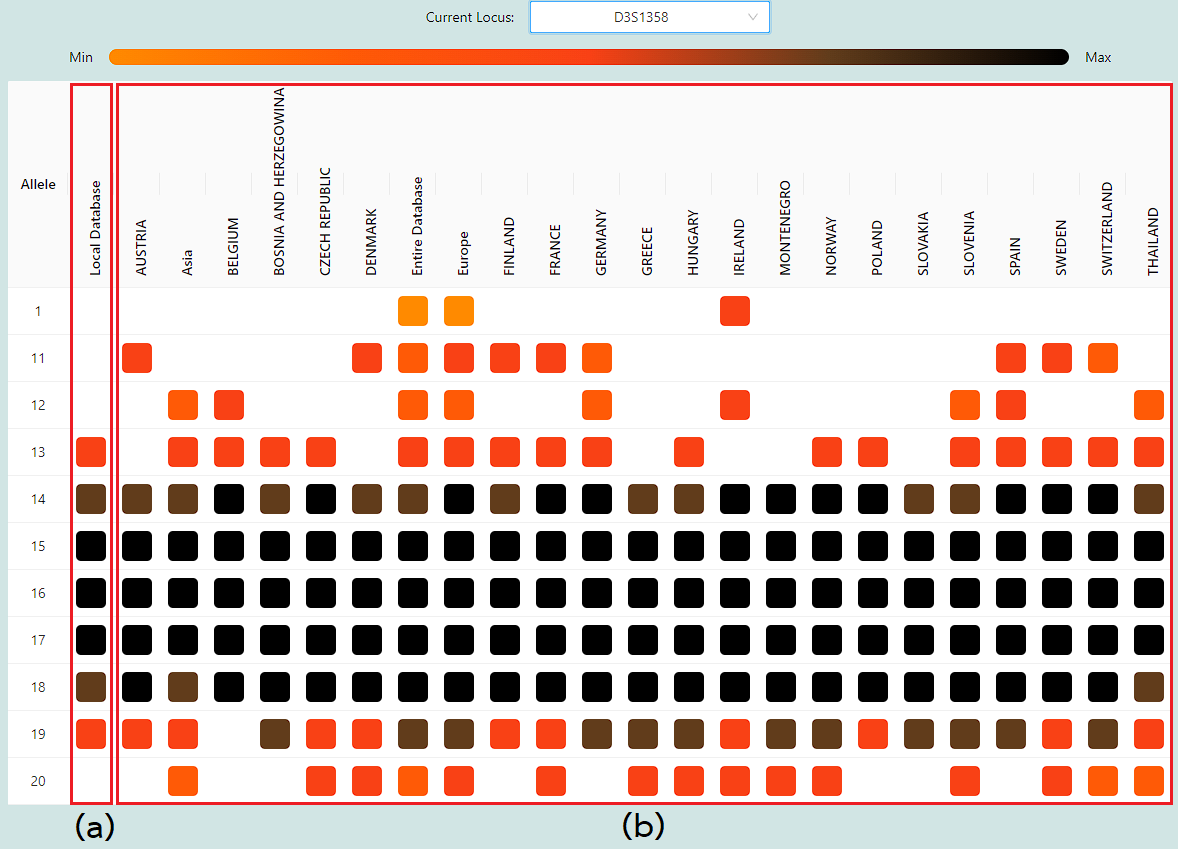

Supplement: S6 Fig — (a) allele frequency calculated from all samples within the STRategy database (b) allele frequency of various countries obtained from STRidER. (TIF) [file pone.0282551.s006.tif]

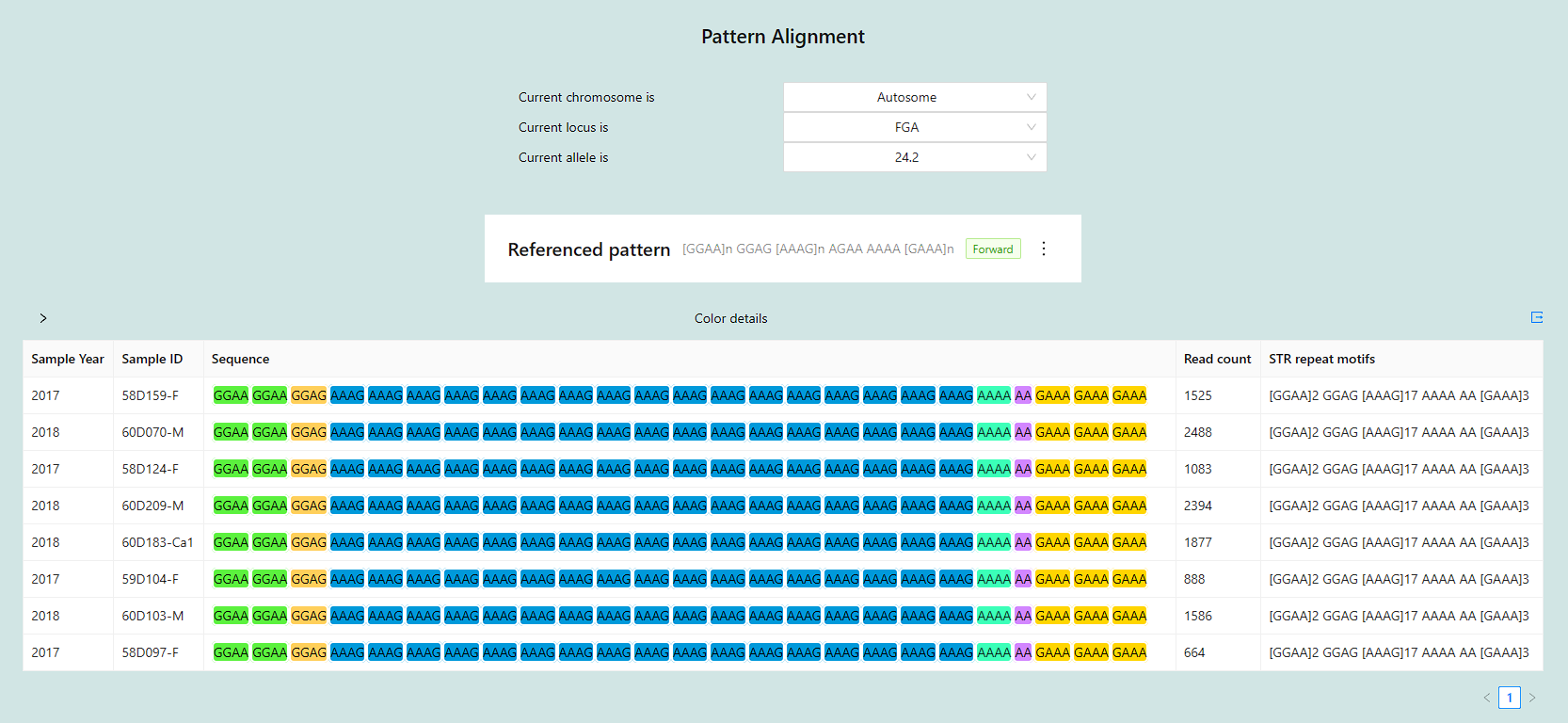

Supplement: S7 Fig — (TIF) [file pone.0282551.s007.tif]

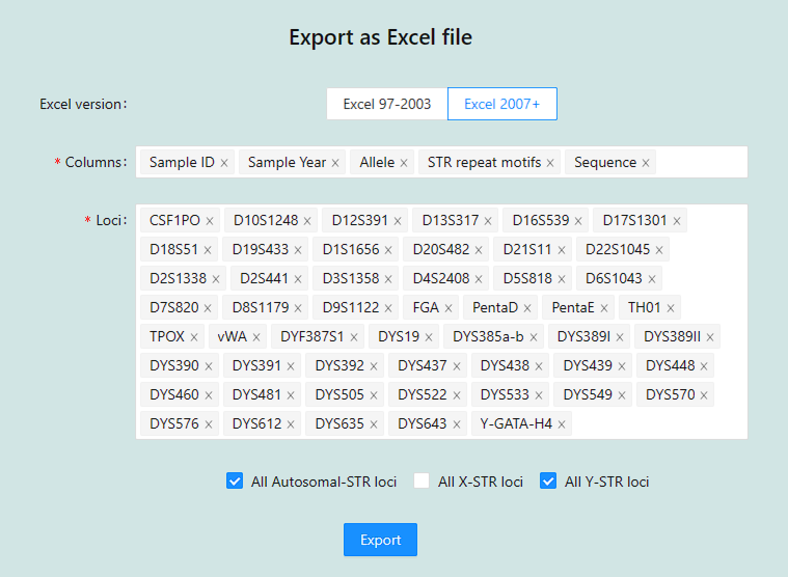

Supplement: S8 Fig — (TIF) [file pone.0282551.s008.tif]

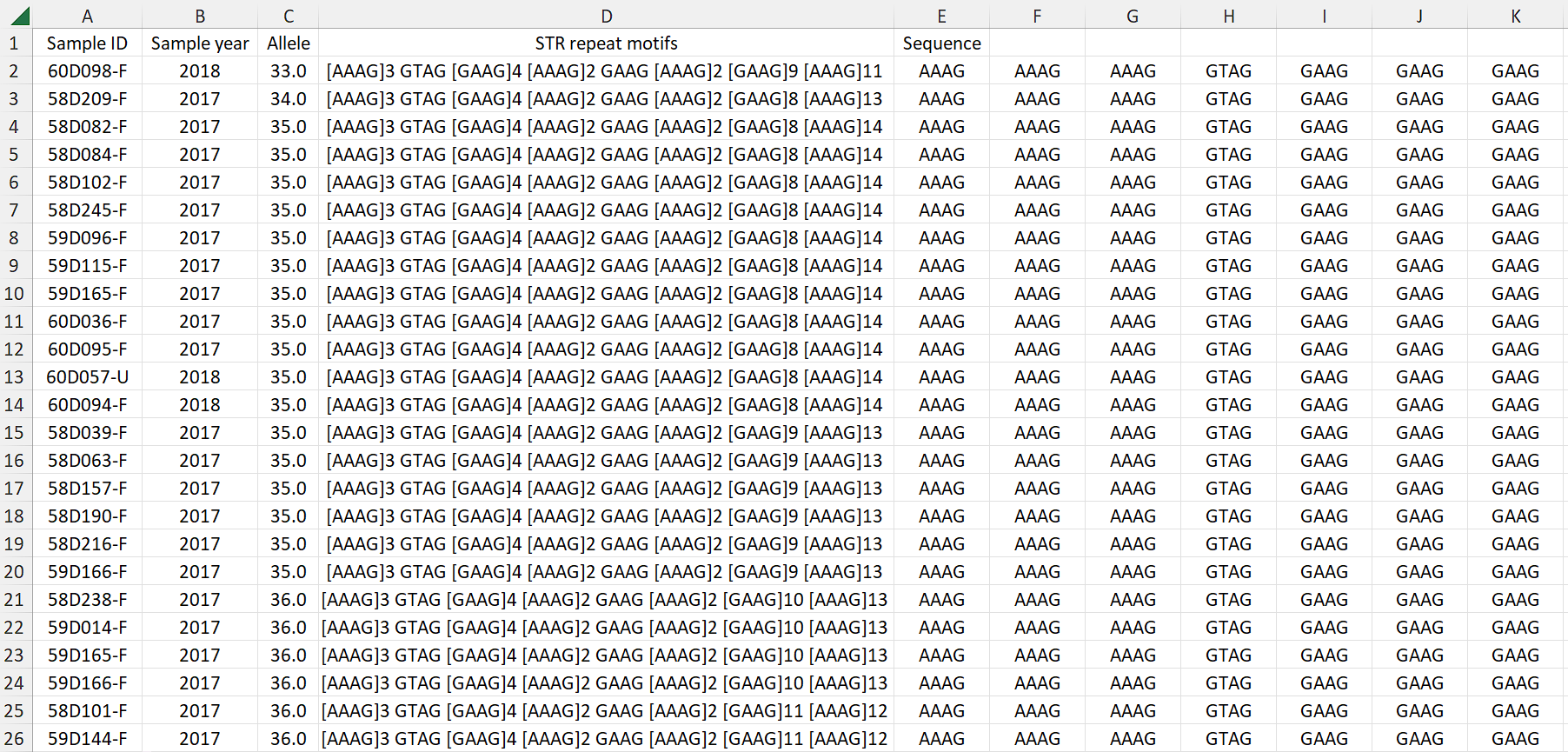

Supplement: S9 Fig — (TIF) [file pone.0282551.s009.tif]

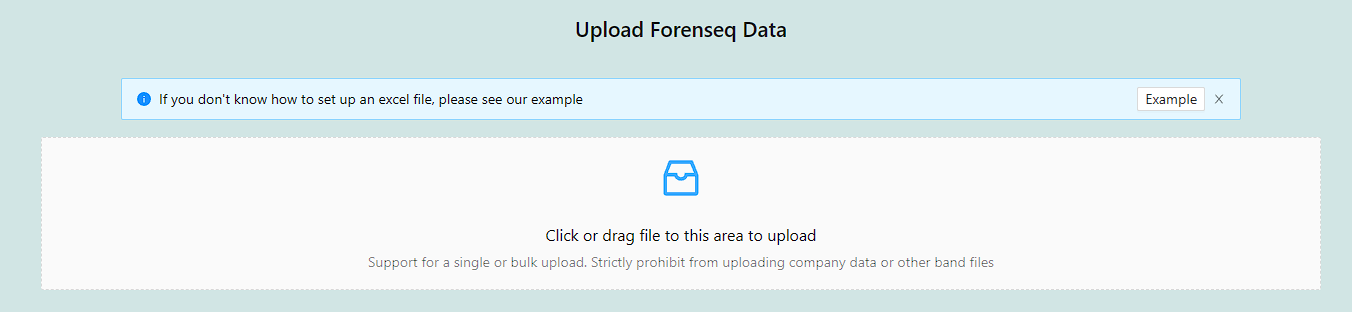

Supplement: S10 Fig — (TIF) [file pone.0282551.s010.tif]

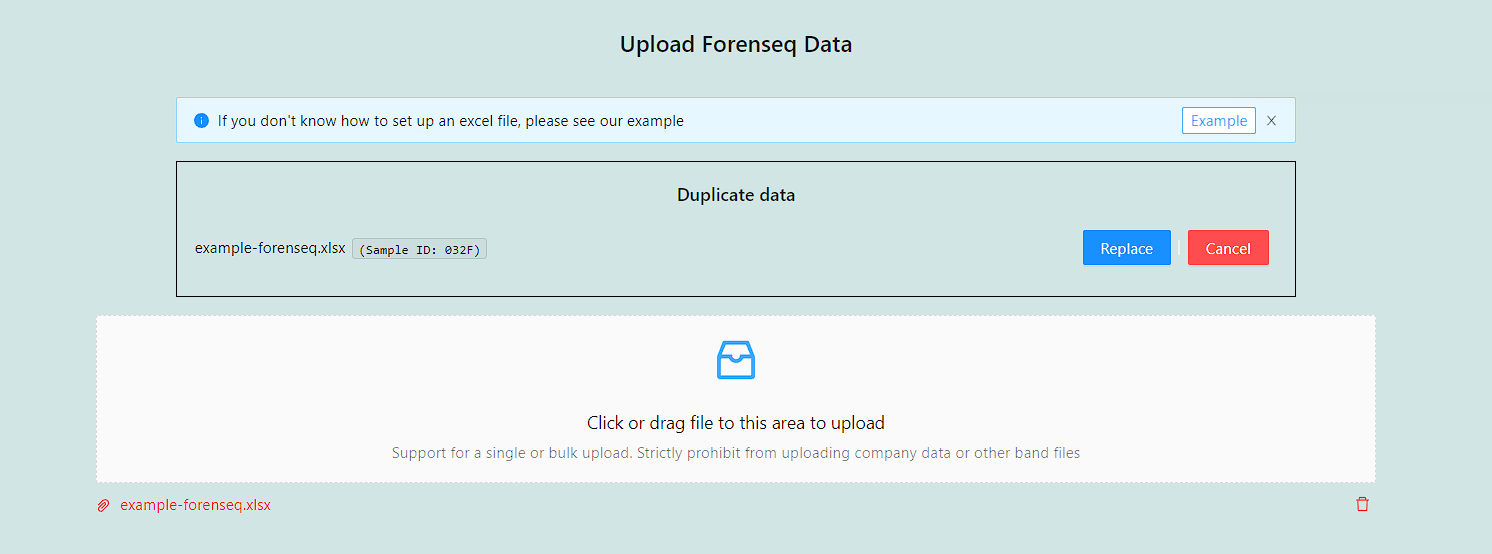

Supplement: S11 Fig — (TIF) [file pone.0282551.s011.tif]

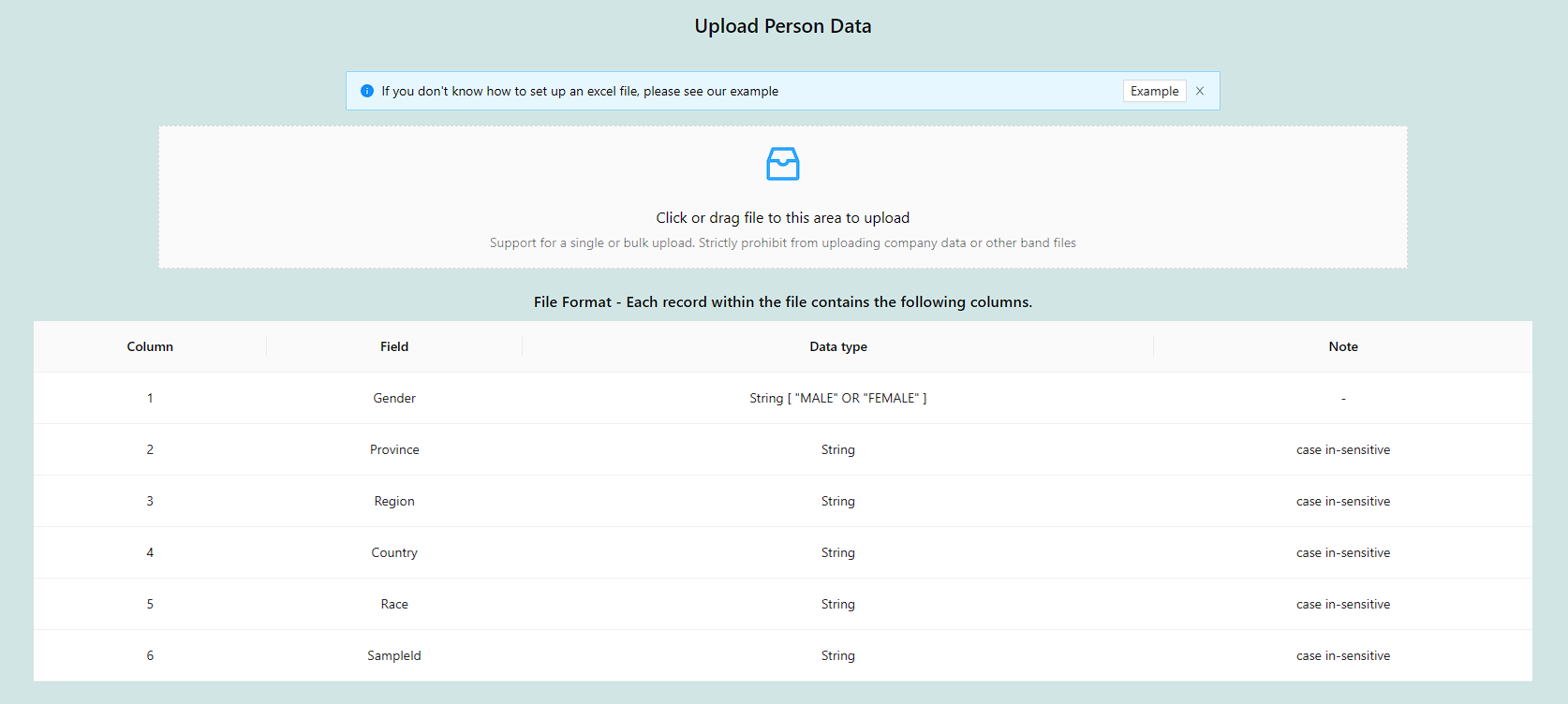

Supplement: S12 Fig — (TIF) [file pone.0282551.s012.tif]

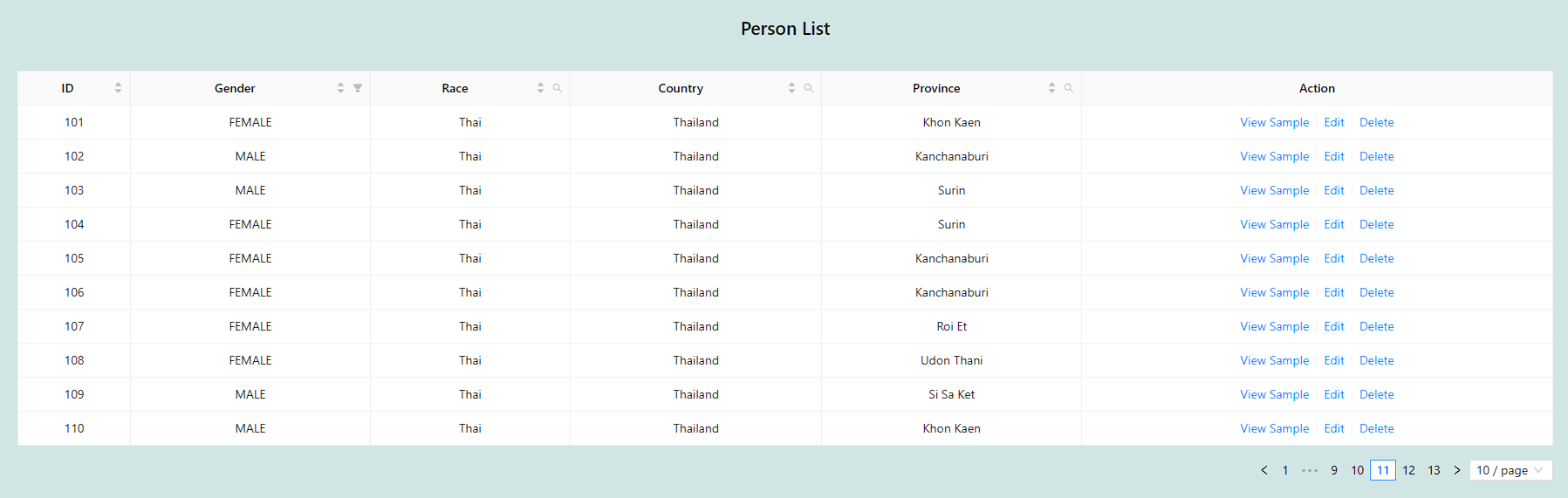

Supplement: S13 Fig — (TIF) [file pone.0282551.s013.tif]

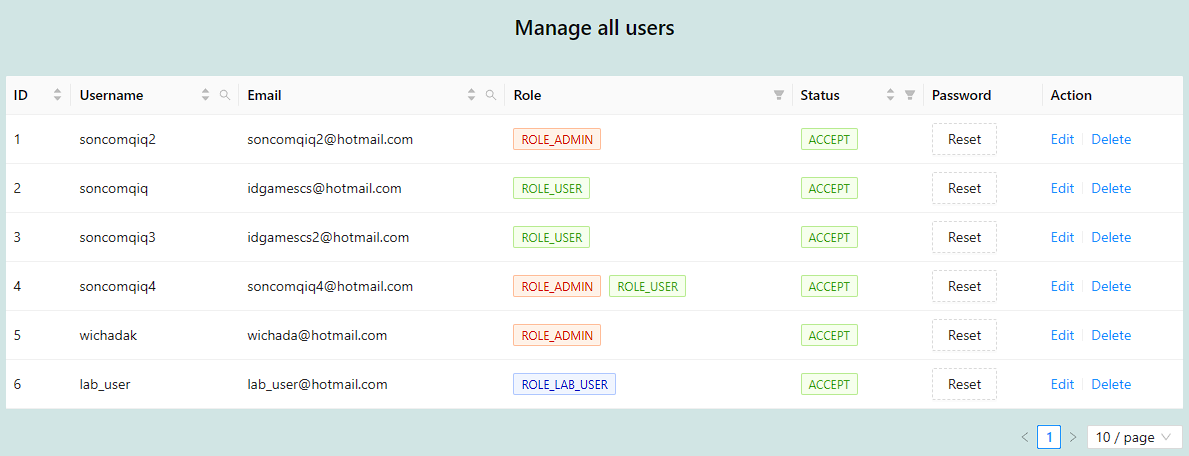

Supplement: S14 Fig — (TIF) [file pone.0282551.s014.tif]

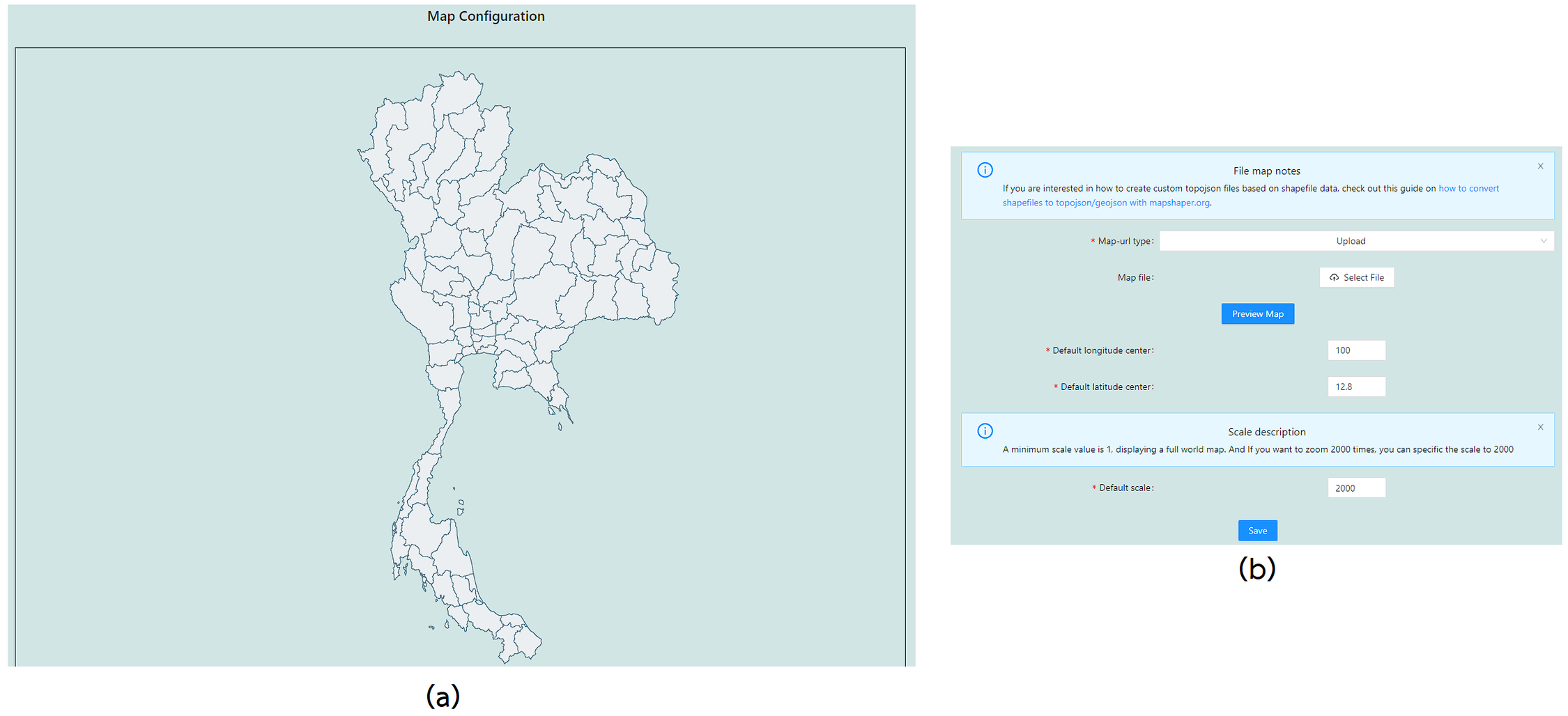

Supplement: S15 Fig — (a) Preview page (b) Configuration panel. (TIF) [file pone.0282551.s015.tif]

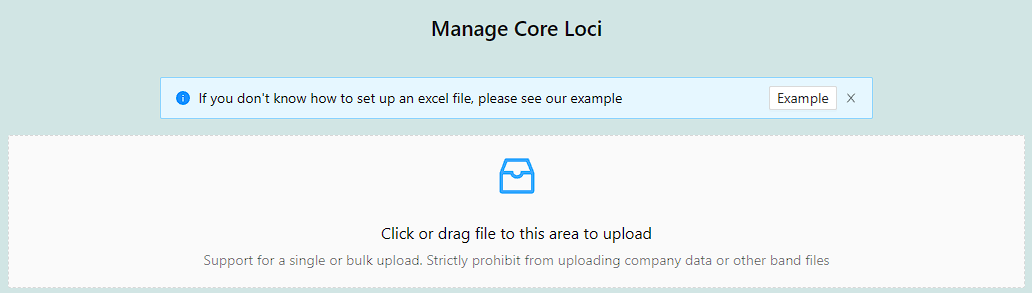

Supplement: S16 Fig — (TIF) [file pone.0282551.s016.tif]

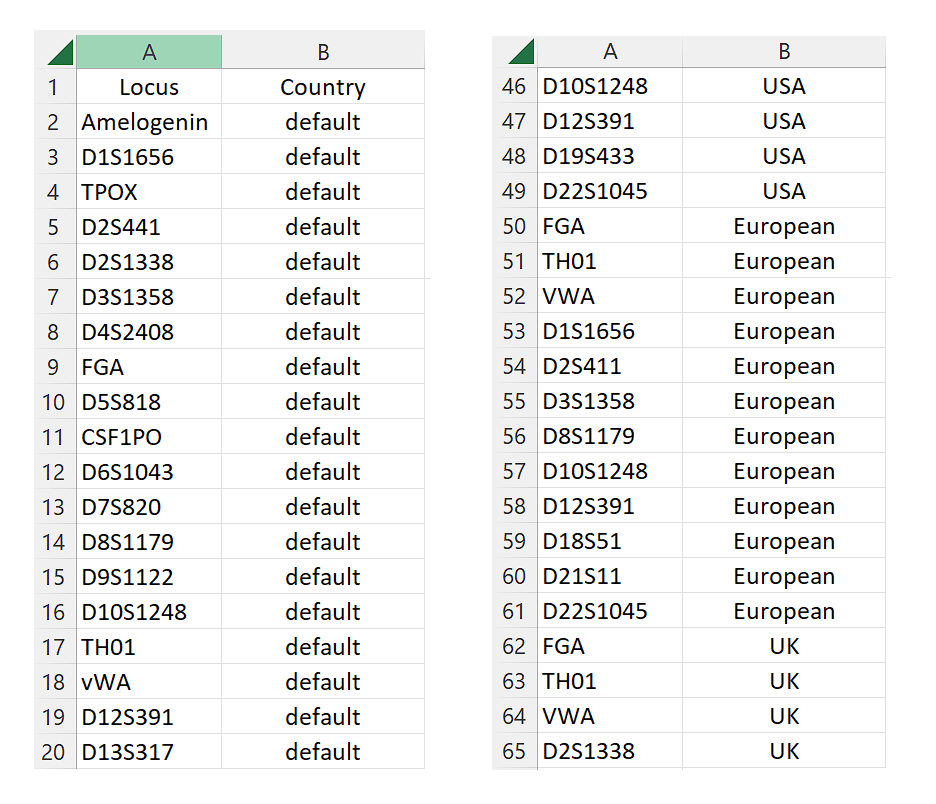

Supplement: S17 Fig — (TIF) [file pone.0282551.s017.tif]

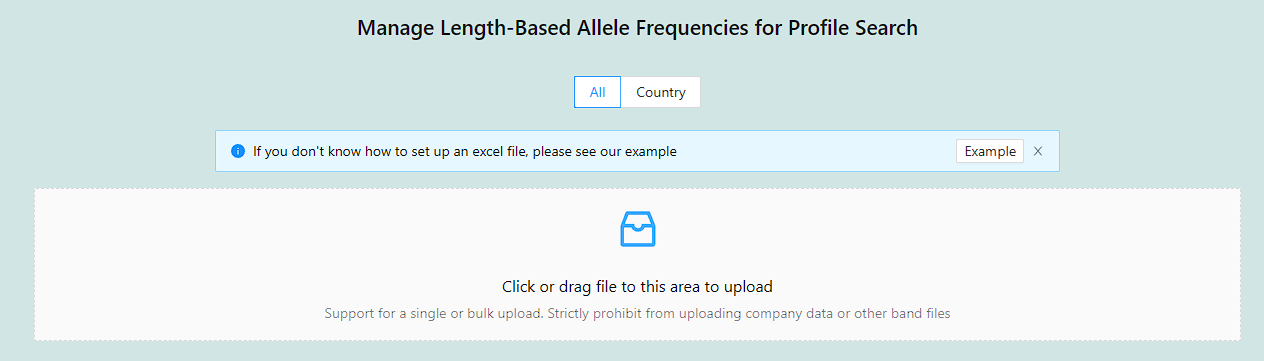

Supplement: S18 Fig — (TIF) [file pone.0282551.s018.tif]

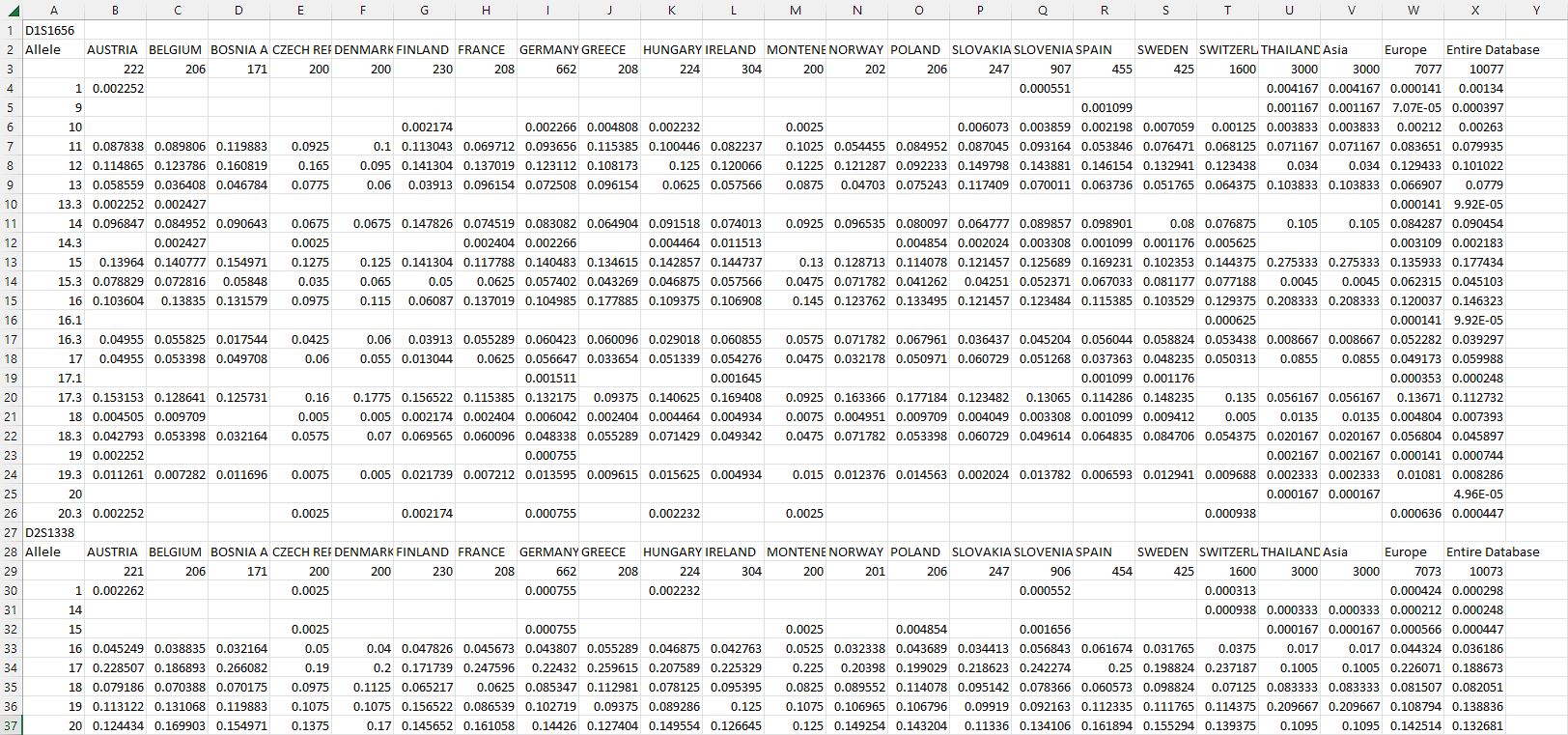

Supplement: S19 Fig — (TIF) [file pone.0282551.s019.tif]

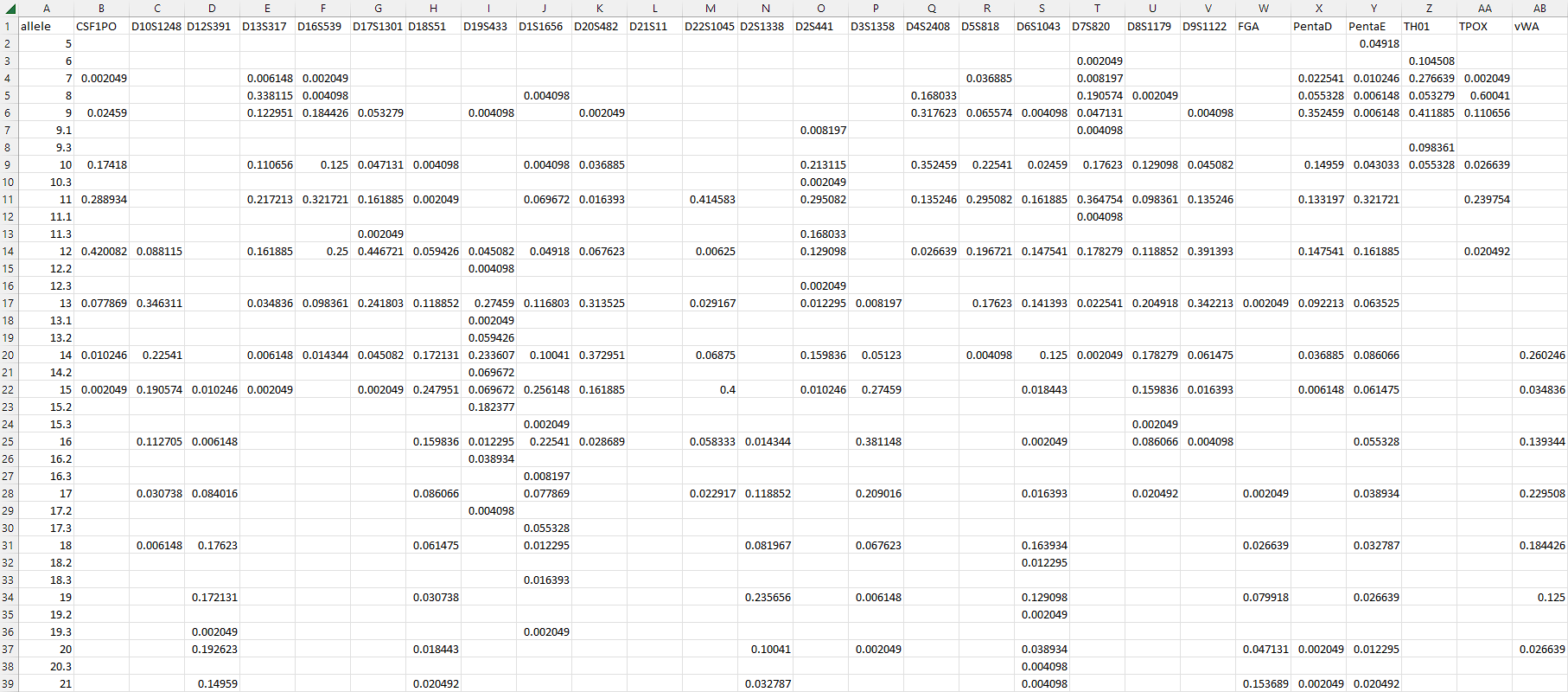

Supplement: S20 Fig — (TIF) [file pone.0282551.s020.tif]

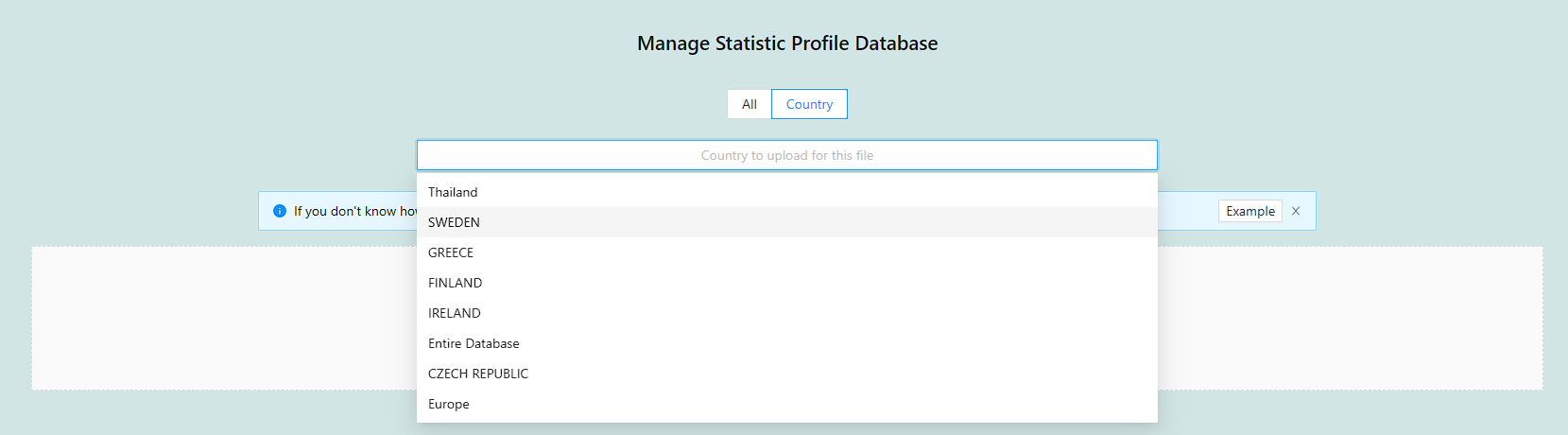

Supplement: S21 Fig — (TIF) [file pone.0282551.s021.tif]

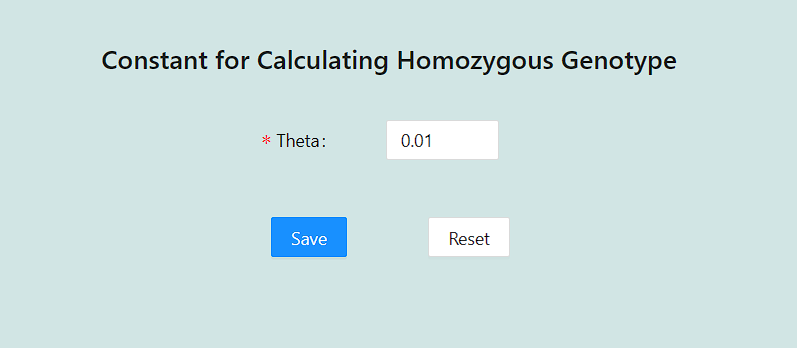

Supplement: S22 Fig — (TIF) [file pone.0282551.s022.tif]

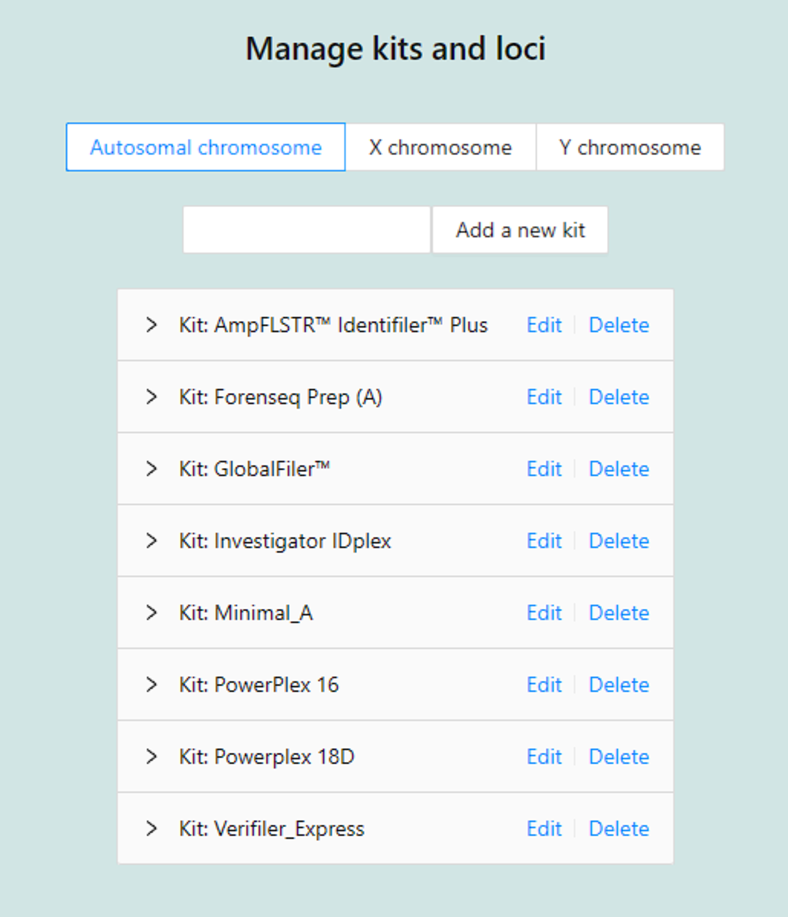

Supplement: S23 Fig — (TIF) [file pone.0282551.s023.tif]

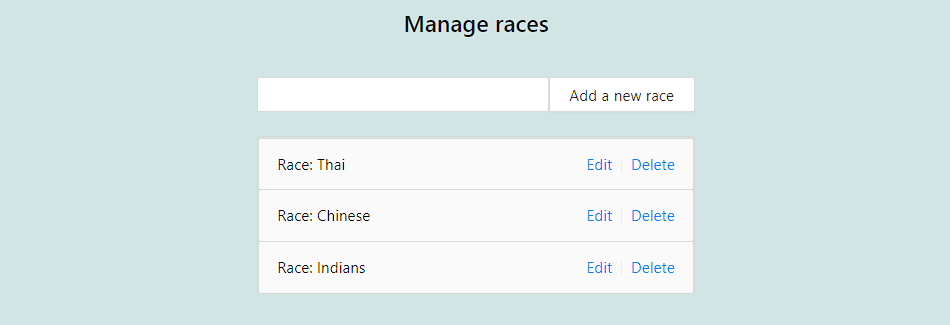

Supplement: S24 Fig — (TIF) [file pone.0282551.s024.tif]

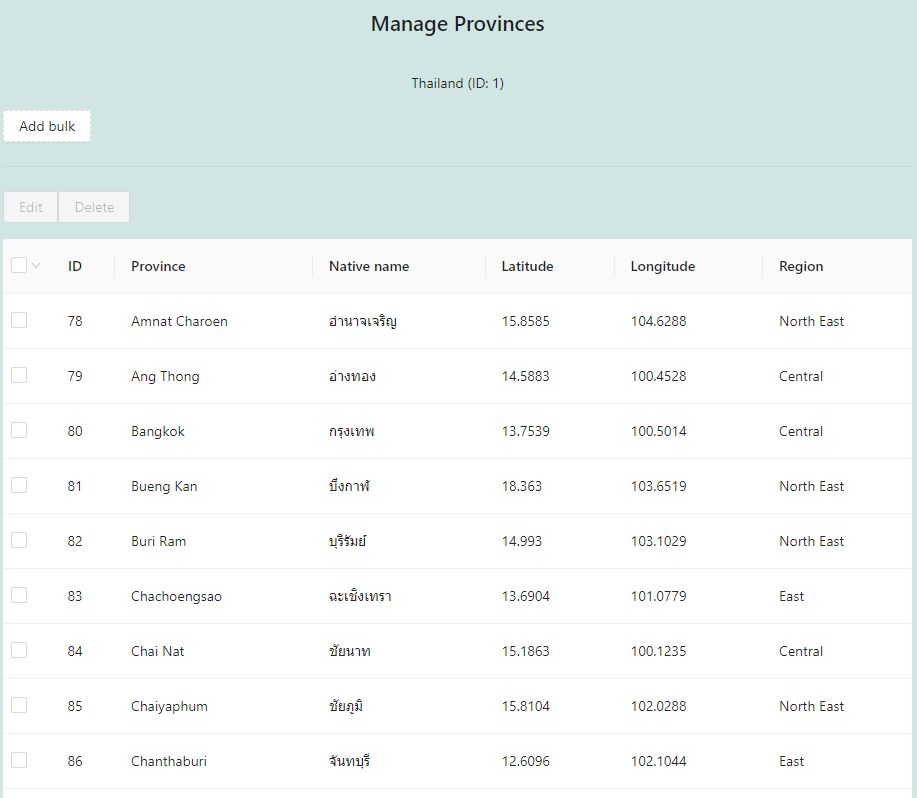

Supplement: S25 Fig — (TIF) [file pone.0282551.s025.tif]

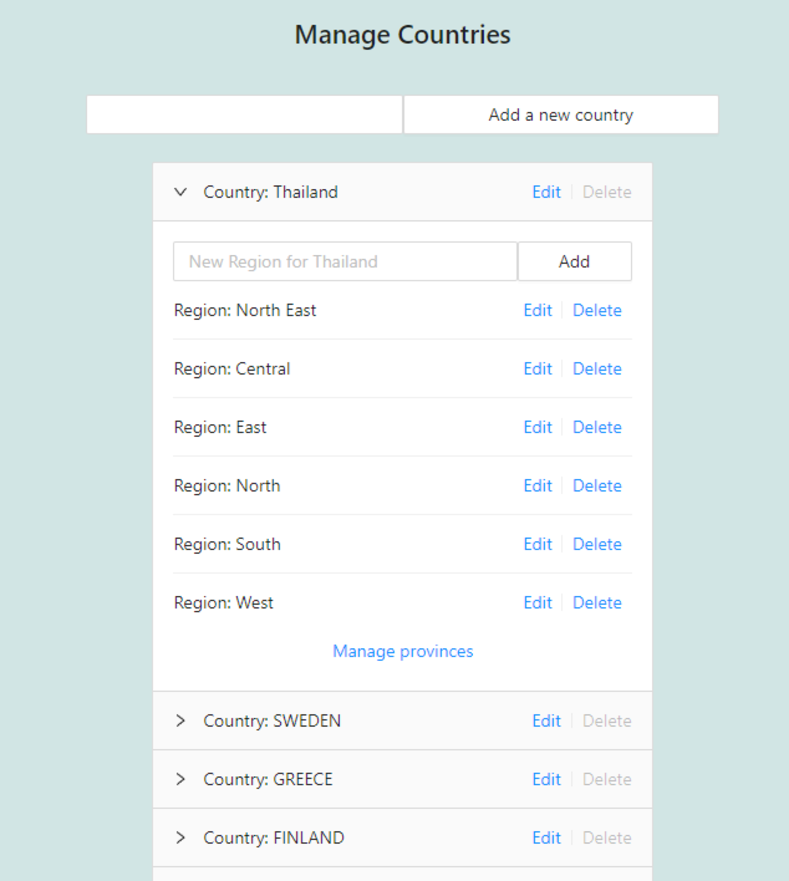

Supplement: S26 Fig — (TIF) [file pone.0282551.s026.tif]

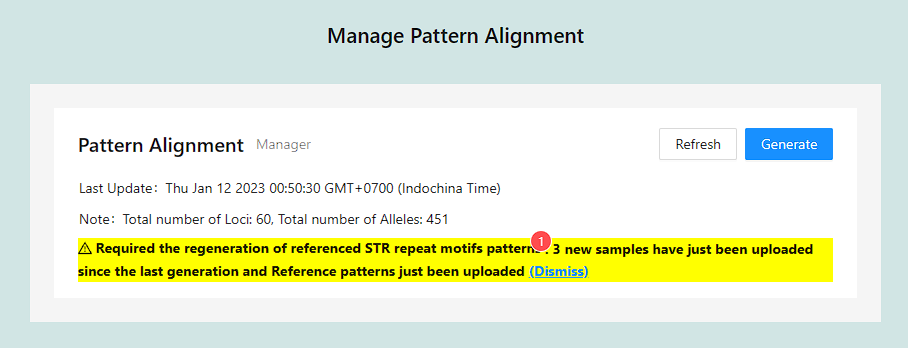

Supplement: S27 Fig — (TIF) [file pone.0282551.s027.tif]

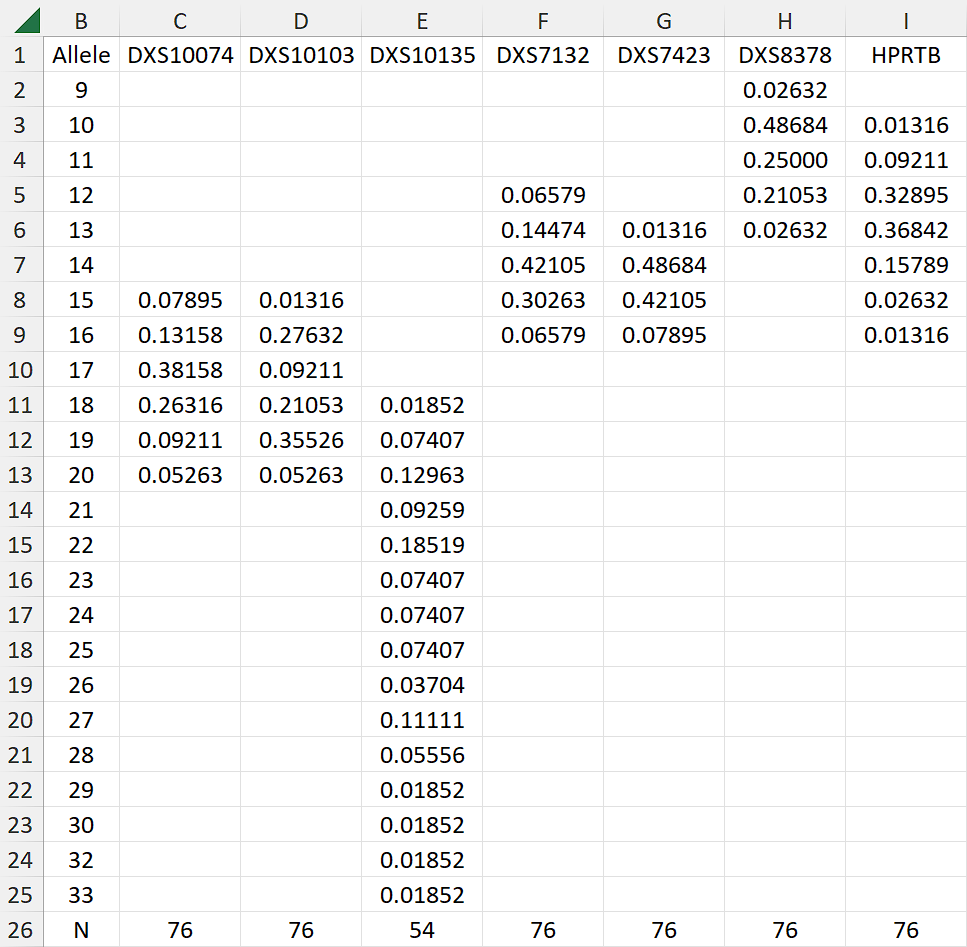

Supplement: S28 Fig — (TIF) [file pone.0282551.s028.tif]

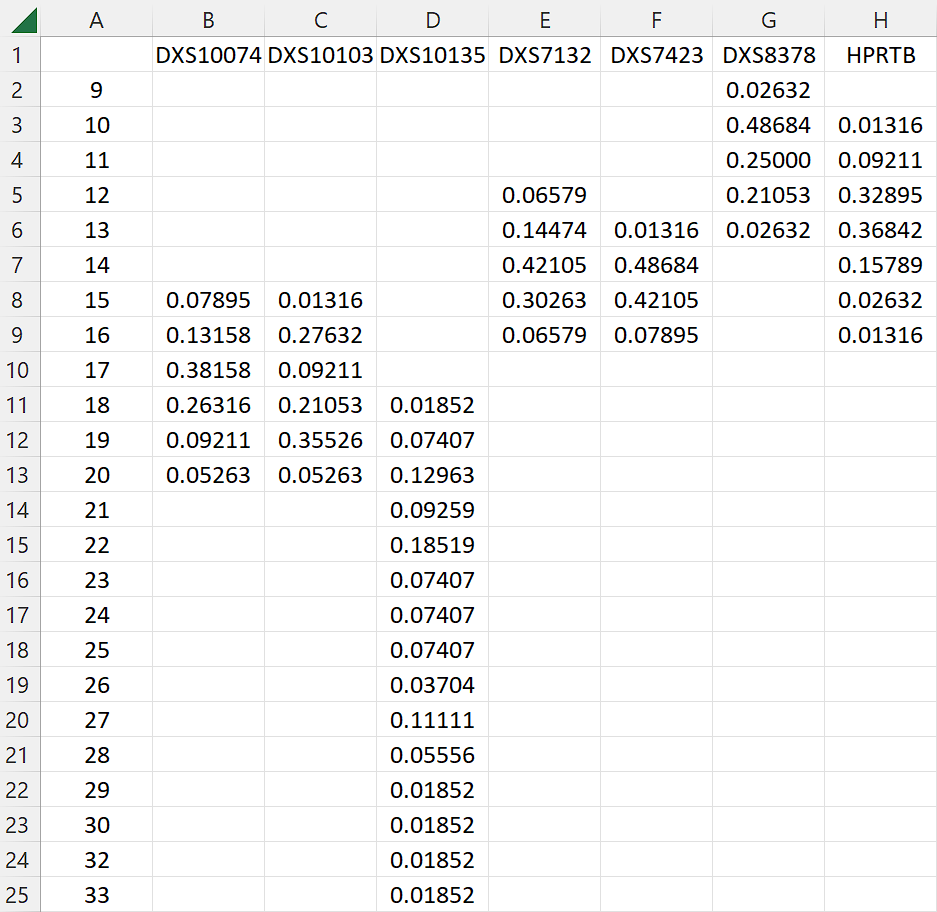

Supplement: S29 Fig — (TIF) [file pone.0282551.s029.tif]

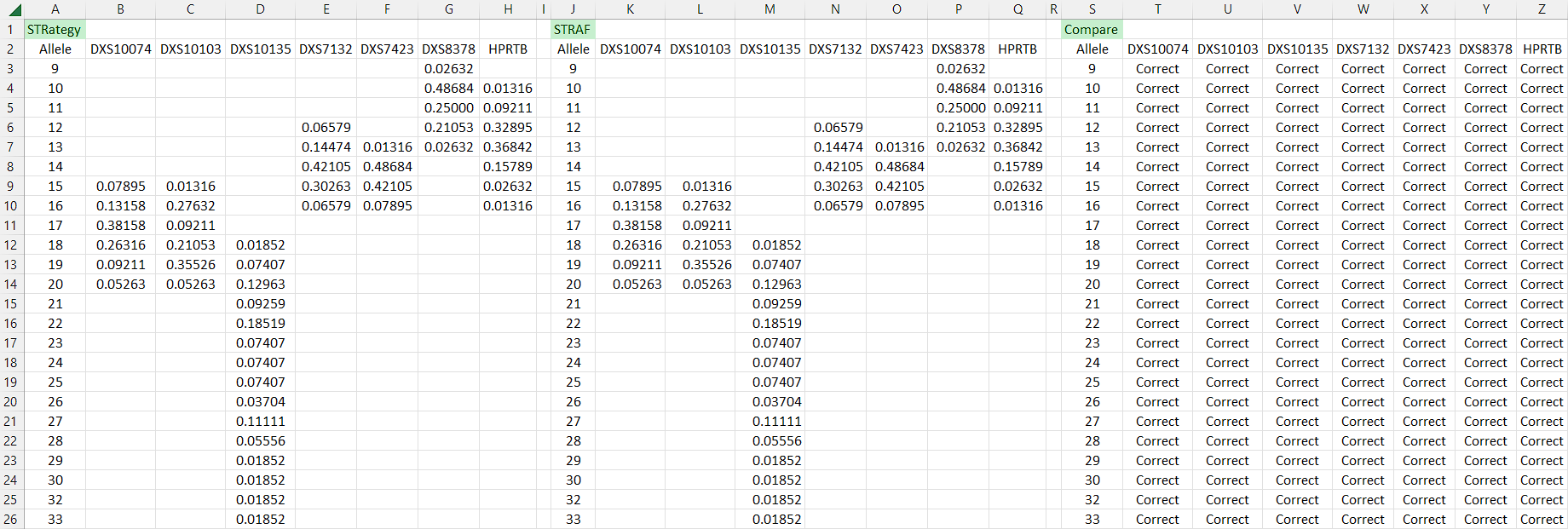

Supplement: S30 Fig — (TIF) [file pone.0282551.s030.tif]

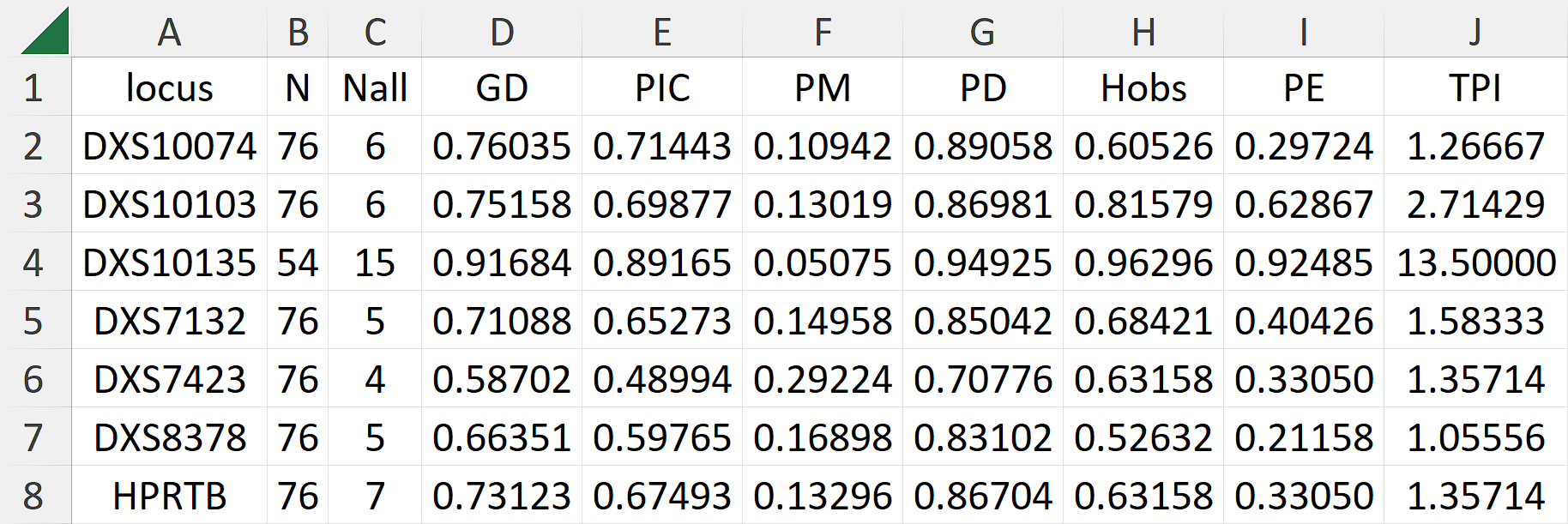

Supplement: S31 Fig — (TIF) [file pone.0282551.s031.tif]

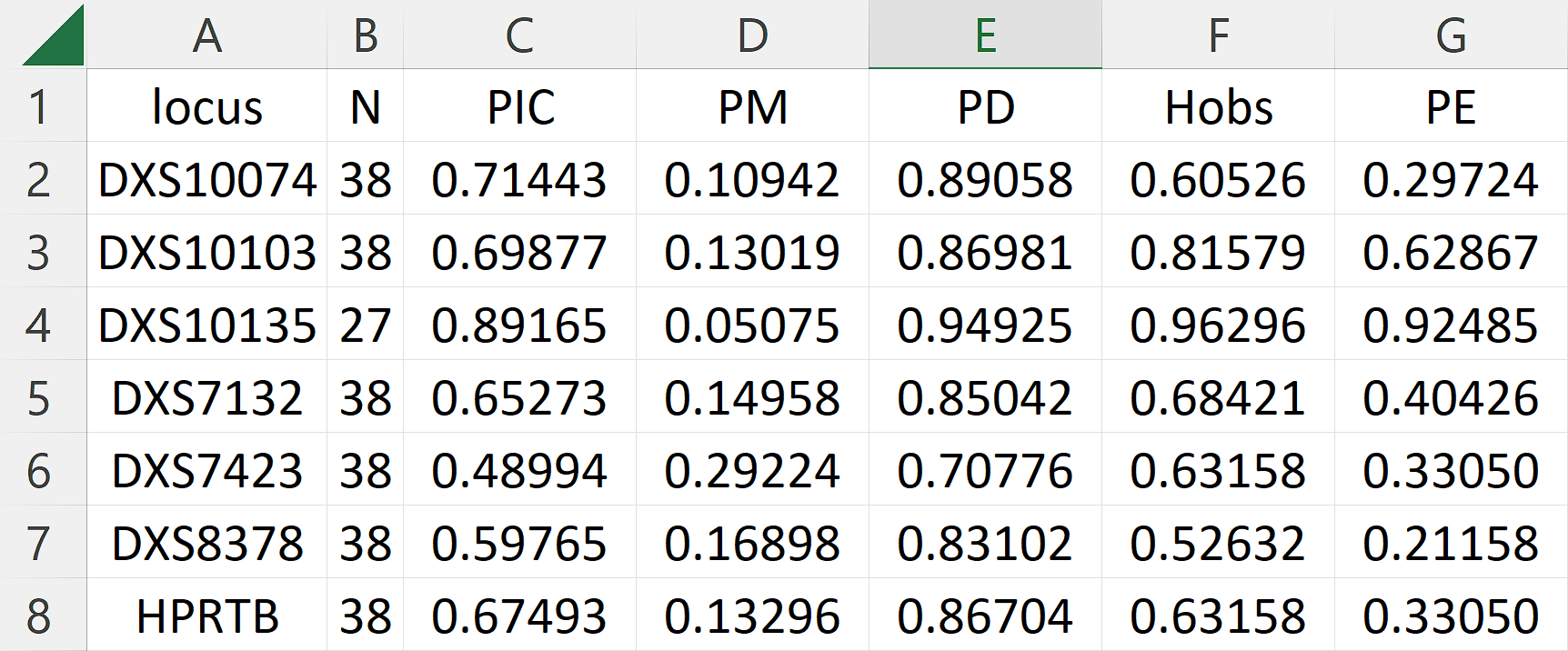

Supplement: S32 Fig — (TIF) [file pone.0282551.s032.tif]

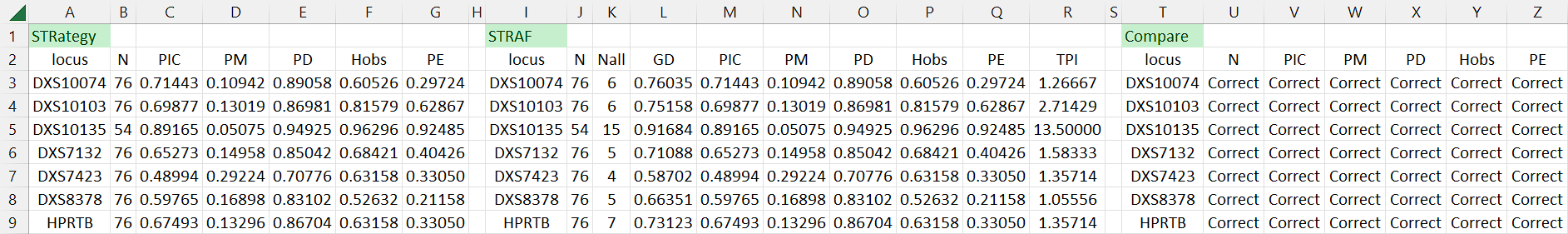

Supplement: S33 Fig — (TIF) [file pone.0282551.s033.tif]
